# A Negative Feedback Loop That Limits the Ectopic Activation of a Cell Type–Specific Sporulation Sigma Factor of *Bacillus subtilis*

**DOI:** 10.1371/journal.pgen.1002220

**Published:** 2011-09-15

**Authors:** Mónica Serrano, Gonçalo Real, Joana Santos, Jorge Carneiro, Charles P. Moran, Adriano O. Henriques

**Affiliations:** 1 Instituto de Tecnologia Química e Biológica, Universidade Nova de Lisboa, Oeiras, Portugal; 2 Instituto Gulbenkian de Ciência, Oeiras, Portugal; 3 Department of Microbiology and Immunology, Emory University School of Medicine, Atlanta, Georgia, United States of America; Agency for Science, Technology, and Research, Singapore

## Abstract

Two highly similar RNA polymerase sigma subunits, σ^F^ and σ^G^, govern the early and late phases of forespore-specific gene expression during spore differentiation in *Bacillus subtilis*. σ^F^ drives synthesis of σ^G^ but the latter only becomes active once engulfment of the forespore by the mother cell is completed, its levels rising quickly due to a positive feedback loop. The mechanisms that prevent premature or ectopic activation of σ^G^ while discriminating between σ^F^ and σ^G^ in the forespore are not fully comprehended. Here, we report that the substitution of an asparagine by a glutamic acid at position 45 of σ^G^ (N45E) strongly reduced binding by a previously characterized anti-sigma factor, CsfB (also known as Gin), in vitro, and increased the activity of σ^G^ in vivo. The N45E mutation caused the appearance of a sub-population of pre-divisional cells with strong activity of σ^G^. CsfB is normally produced in the forespore, under σ^F^ control, but *sigGN45E* mutant cells also expressed *csfB* and did so in a σ^G^-dependent manner, autonomously from σ^F^. Thus, a negative feedback loop involving CsfB counteracts the positive feedback loop resulting from ectopic σ^G^ activity. N45 is invariant in the homologous position of σ^G^ orthologues, whereas its functional equivalent in σ^F^ proteins, E39, is highly conserved. While CsfB does not bind to wild-type σ^F^, a E39N substitution in σ^F^ resulted in efficient binding of CsfB to σ^F^. Moreover, under certain conditions, the E39N alteration strongly restrains the activity of σ^F^ in vivo, in a *csfB*-dependent manner, and the efficiency of sporulation. Therefore, a single amino residue, N45/E39, is sufficient for the ability of CsfB to discriminate between the two forespore-specific sigma factors in *B. subtilis*.

## Author Summary

Positive auto-regulation of a transcriptional activator during cell differentiation or development often allows the rapid and robust deployment of cell- and stage-specific genes and the routing of the differentiating cell down a specific path. Positive auto-regulation however, raises the potential for inappropriate activity of the transcription factor. Here we unravel the role of a previously characterized anti-sigma factor, CsfB, in a negative feedback loop that prevents ectopic expression of the sporulation-specific sigma factor σ^G^ of *Bacillus subtilis*. σ^G^ is activated in the forespore, one of the two chambers of the developing cell, at an intermediate stage in spore development. Once active, a positive feedback loop allows the rapid accumulation of σ^G^. Synthesis of both σ^G^ and CsfB is under the control of the early forespore regulator σ^F^, and CsfB may help prevent the premature activity of σ^G^ in the forespore. However, CsfB is also produced under σ^G^ control in non-sporulating cells, setting a negative feedback loop that we show limits its ectopic activation. We further show that an asparagine residue conserved among σ^G^ orthologues is critical for binding and inhibition by CsfB, whereas the exclusion of asparagine from the homologous position in σ^F^ confers immunity to CsfB.

## Introduction

**Figure 1:**
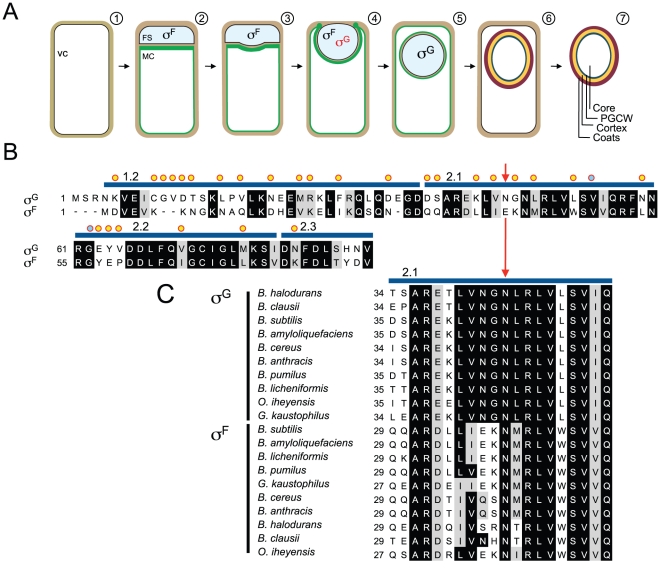
Segregation of σ^F^ and σ^G^ activities and mutagenesis of *sigG*. Panel A shows the main stages in sporulation and the temporal windows of activity of σ^F^ and σ^G^ in the forespore. Shown is (1) a pre-divisional cell, (2) a cell that has completed asymmetric division forming a forespore (FS) and a mother cell compartment (MC), (3, 4) a cell during and (5) after engulfment completion, (6) the following assembly of the spore protective layers and (7) release of the mature spore upon mother cell lysis. The mother cell membranes are colored in green. σ^F^ is activated in the forespore soon after polar division, and transcribes the *sigG* gene, but σ^G^ only becomes active following engulfment completion. Red and black denote inactive and active σ^G^, respectively. Panel B shows an alignment between the N-terminal regions of σ^G^ and σ^F^ from *B. subtilis*. Residues in σ^G^ that were subject to mutagenesis are indicated by yellow circles or, for N45, by a red arrow. The blue lines above the sequence delimit conserved regions 1.2 through 2.3. Residues previously implicated in binding of SpoIIAB to σ^F^ are marked with a blue circle. Panel C shows the alignment of region 2.1 of *B. subtilis* σ^G^ (highlighting residue N45 with a red arrow) with the same region of σ^G^ and σ^F^ from other, selected, *Bacillus* species. The alignments (B and C) were prepared using ClustalW (http://www.ch.embnet.org/software/ClustalW.html). Black and grey backgrounds highlight identical or conserved residues, respectively.

When cells of *Bacillus subtilis* enter stationary phase and face severe nutrient depletion, they may embark into a developmental pathway that results in the production of a dormant, highly resistant endospore [1]. Sporulation involves the asymmetric division of the rod-shape cell into a smaller forespore, the future spore, and a larger mother cell. Soon after asymmetric cell division, the mother cell engulfs the forespore, eventually releasing it as a free protoplast within its cytoplasm. Following engulfment completion, the forespore becomes encased in a series of protective layers after which it is released into the environment through lysis of the mother cell [1]. Underlying the differentiation process are mother cell- and forespore-specific programs of gene expression administered by a cascade of cell type-specific RNA polymerase sigma factors. σ^F^ and σ^E^ govern the initial stages in development in the forespore and in the mother cell, respectively. At late stages of development, σ^F^ is replaced by σ^G^ (Figure 1A), whereas σ^K^ replaces σ^E^. The sporulation-specific sigma factors are produced prior to their period of activity, and maintained inactive until the successful conclusion of key morphological events during development. Both σ^F^ and σ^E^ are synthesized in the predivisional cell. Proper septation is a prerequisite for the activation of σ^F^ in the forespore and soon after a signaling pathway initiated by σ^F^ leads to the activation of σ^E^ in the mother cell. Likewise, synthesis of σ^G^ and σ^K^ is initially driven by σ^F^ and σ^E^, respectively. However, σ^E^-dependent gene expression is required for the activation of σ^G^ following engulfment completion and when active, σ^G^ initiates a signaling pathway that causes the activation of σ^K^ ([1]–[3] see also below). The double responsiveness of the cell-type specific σ factors to proper morphogenesis and to intercompartmental signaling pathways effectively links the forespore and mother cell programs of gene expression and keeps gene expression in close register with the course of morphogenesis. Importantly, proper timing of sigma factor activation is essential for the fidelity of the developmental process [reviewed by [1]–[3]].

This study addresses the mechanisms involved in the regulation of the activity of σ^G^. Productive transcription of the *sigG* gene (coding for σ^G^) is controlled by σ^F^ [4], [5]. However, *sigG* is not transcribed as soon as σ^F^ becomes active. The delay appears to result from an as yet poorly understood dependency of *sigG* transcription upon the activity of σ^E^ in the mother cell [6], [7]. σ^G^ can be detected in the forespore towards the end of the engulfment sequence, but its window of activity begins only after engulfment completion. Activity of σ^G^ requires the assembly of a novel type of secretion system formed by eight mother cell proteins (AA through AH) coded for by the σ^E^-controlled *spoIIIA* operon, and by the forespore-specific, σ^F^-controlled protein SpoIIQ [8]–[14], with the assistance of the membrane protein translocase SpoIIIJ [8],[15]–[18]. The SpoIIIA-SpoIIQ complex spans the intermembrane space that separates the forespore and the mother cell establishing a direct connection between the cytoplasm of the two cells [8], [10], [14], [19]. Recent work has lead to the concept that the channel acts as a feeding tube, maintaining the potential for macromolecular synthesis when the forespore becomes isolated from the external medium [9]. This model brings the important implication that the activation of σ^G^ in engulfed forespores does not necessarily involve counteracting a specific inhibitor or inhibitors of σ^G^. However, once active, σ^G^ recognizes its own promoter, creating a positive feedback loop that causes its levels to increase rapidly [4], [5]. This autoregulatory effect implies the tight regulation of σ^G^ activation so that its normal timing and cell specificity are both observed, and raises questions regarding the mechanisms that prevent activation of the positive feedback in the forespore prior to engulfment completion, or in non-sporulating cells.

Three negative regulators of σ^G^ are known, the LonA protease, and the anti-sigma factors SpoIIAB and CsfB [12], [20]–[22]. LonA, an ATP-dependent serine protease, acts mainly to prevent inappropriate activity of σ^G^ under culture conditions in which sporulation is not favored [22]–[24]. During sporulation LonA may only be active in the mother cell, because its forced expression in the forespore strongly interferes with sporulation [23], [24]. Genetic and biochemical experiments have shown that SpoIIAB, the anti-sigma factor that maintains σ^F^ inactive prior to the asymmetric division of sporulating cells, also binds to σ^G^ [12], [24]–[26]. However, while SpoIIAB contributes to the inactivity of σ^G^ under non-sporulation conditions and in the mother cell during sporulation it does not play a critical role in the negative regulation of σ^G^ in the forespore [8], [21], [24]). A third negative regulator of σ^G^ is CsfB (also known as Gin), a novel type of Zn^2+^ anti-sigma factor [20], [27], [28]. CsfB combines two properties expected for a factor capable of inhibiting σ^G^ prior to engulfment completion: specificity for σ^G^ (unlike SpoIIAB, CsfB does not binds to σ^F^) and its early presence in the forespore compartment [20], [28], [29]. However, although one group initially proposed that CsfB had a key role in the negative regulation of σ^G^ in the pre-engulfed forespore [20], other groups did not observe massive premature activation of σ^G^ in the forespore upon deletion of the *csfB* gene [8], [27].

While the auto regulatory nature of σ^G^ seems to justify the existence of multiple negative regulators, none of the known regulators *per se*, seems to have a decisive role in preventing activation of the σ^G^ positive feedback loop. Because σ^F^ and σ^G^ are very similar proteins, we reasoned that the residues in which the two proteins differ could hold the key to their differential regulation. We changed all the residues within conserved regions 1.2 through the beginning of region 2.3 of σ^G^ in which it differs from σ^F^ to the residue found in this latter protein. We report the identification of a mutation (N45E) that reduces binding of CsfB to σ^G^ in vivo and in vitro. The mutation also results in the appearance of a population of stationary phase cells in which σ^G^ becomes active. We show that σ^G^ drives expression of *csfB* in these cells, setting-up a negative feedback loop that limits its activation across the population.

We further show the importance of N45 in σ^G^ and its equivalent in σ^F^ (E39), in the different responsiveness of the two forespore-specific sigma factors to CsfB. While unable to bind to wild type σ^F^, CsfB interacts with a form of σ^F^ in which E39 is replaced by an N residue, found in the corresponding position of σ^G^ (N45). Importantly, we show that the E39N substitution can strongly inhibit the forespore-specific activity of σ^F^ and the efficiency of sporulation. Thus, a single amino acid residue allows CsfB to discriminate between the two highly similar forespore-specific sigma factors. This property is likely to be widespread, because N45 is invariant in *Bacillus* orthologues of σ^G^, while with a single exception N is excluded from the equivalent position in the σ^F^ proteins of the same species.

## Results

### A mutation in conserved region 2.2 that increases the activity of σ^G^

**Figure 2:**
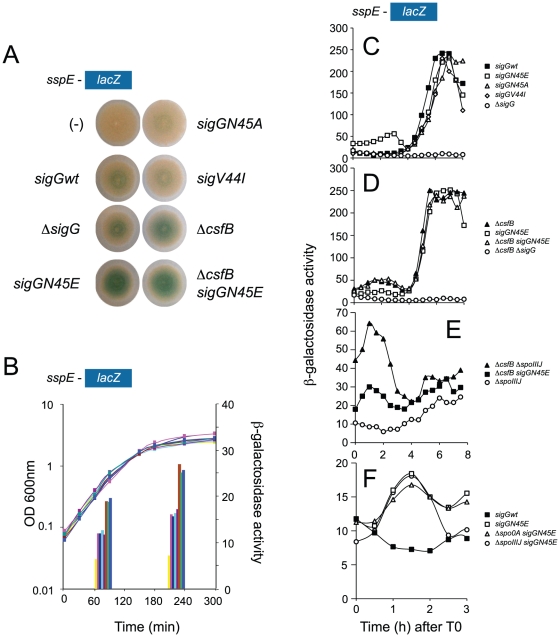
Identification of mutants with increased activity of σ^G^. Panel A illustrates a colony screening for enhanced β-galactosidase production from the σ^G^-controlled P*_sspE_*-*lacZ* reporter fusion on LB plates containing X-Gal. Panel B shows the quantitative analysis of enzyme activity for the same strains shown in A, on liquid LB cultures at mid-log and at the onset of stationary phase. The following strains are shown (Table S1 shows the complete genotype of all strains): wild type background (pink in panel B), *sigGN45E* (brown in panel B), *sigGN45A* (light blue in panel B), *sigGV44I* (purple in panel B), Δ*csfB* (green in panel B), and Δ*csfB sigGN45E* (blue in panel B). Controls for background levels are the wild type MB24 bearing no reporter fusion (“−” symbol in panel A, yellow bars in panel B), and a strain carrying the P*_sspE_-lacZ* fusion in conjunction with a *sigG* deletion mutation (dark blue in panel B). In panels C, D, E, and F the activity of wild type σ^G^ or its mutant forms σ^GN45A^, σ^GN45E^, or σ^GV44I^ was examined during sporulation in DSM medium in a wild type background or in strains carrying mutations in genes known to control the initiation of sporulation or to influence the activity of wild type σ^G^. All strains used in this analysis carry the σ^G^-controlled P*_sspE_-lacZ* fusion. Panel C, shows the activity profile for wild type σ^G^, and for σ^GN45E^, σ^GN45A^, or σ^GV44I^ in an otherwise wild type background. Residual expression of P*_sspE_*-*lacZ* in a Δ*sigG* mutant is included for reference. Panel D compares the activity of wild type σ^G^ in a Δ*csfB* mutant to the activity of σ^GN45E^ in a wild type background or in a Δ*csfB* mutant. Expression of P*_sspE_*-*lacZ* in a Δ*sigG* Δ*csfB* mutant is also represented. Panel E compares the activity of wild type σ^G^ in a Δ*spoIIIJ* mutant and a Δ*spoIIIJ* Δ*csfB* double mutant to that of σ^GN45E^ in a Δ*spoIIIJ* mutant. Panel F shows the activity σ^GN45E^ in a wild type background, or in the absence of *sigF* or *spo0A* (AH6626, open triangles). The activity of wild type σ^G^ in a wild type background is shown for reference. The various strains were grown in sporulation medium (DSM) and sampled at hourly intervals after T0 (denoting the end of the logarithmic phase of growth). β-galactosidase activity is shown in Miller Units. In all cases, the various forms of σ^G^ are produced from the wt or *sigG* mutant alleles present at the *sigG* locus. Panels C-F show the results of representative experiments, which in all cases were conducted independently at least three times.

Since σ^F^ is active in the forespore in a temporal window when σ^G^ is kept inactive (Figure 1A), we reasoned that we would be able to find one or more substitutions that would render σ^G^ prematurely active. We initiated this study by changing most of the residues within regions 1.2 and 2.1 through the beginning of region 2.3 of σ^G^ that differed from σ^F^ to the amino acid found at the equivalent position in this latter protein (Figure 1B). The mutations were generated in vitro and transferred by congression to the *sigG* locus (see the Materials and Methods section). We then screened for mutants exhibiting elevated levels of σ^G^ -directed gene expression under non-sporulation conditions (during growth in LB) as these conditions previously led to the identification of two negative regulators of σ^G^ [21], [22]. This is possible because active σ^G^ utilizes its own promoter, leading to the establishment of a positive auto regulatory loop that reinforces its activity [5]. We found a single substitution at codon 45 of the *sigG* gene, an asparagine to a glutamic acid (henceforth N45E) that increased the activity of σ^G^ in vivo, as monitored using a fusion of the σ^G^-responsive *sspE* promoter to *lacZ* [5]. The *sspE* gene codes for an abundant small acid-soluble protein required for the efficient return of spores to vegetative growth, and that is normally expressed in the forespore when σ^G^ becomes active [30]–[32]. The N45E mutation stimulated P*_sspE_*-*lacZ* transcription in colonies of cells growing on solid medium as well as in cells growing in liquid medium, where β-galactosidase activity was 2 fold higher in N45E mutant cells than in wild type cells (Figure 2A and 2B). On liquid medium, the activity of σ^GN45E^ was higher when the cells entered stationary phase (Figure 2B). The augmented expression of P*_sspE_*-*lacZ* could be due to increased activity of σ^G^ or alternatively to the titration by σ^GN45E^ of a negative regulator of σ^F^, which at least under some conditions is also able to direct transcription from the *sspE* promoter [5]. To test the model that σ^GN45E^ could titrate an inhibitor of σ^F^, we first examined the effect of two additional point mutations, F91A and Y94A, in region 2.3 of σ^G^ (see Figure S1A). These residues are presumed to play a role in promoter melting (reviewed by [33]), and alanine substitutions at these positions, while allowing the accumulation of σ^G^, inactivate the sigma factor (Text S1 and Figure S1). Importantly, the N45E-stimulated expression of P*_sspE_*-*lacZ* was abolished in a N45E/F91A/Y94A triple mutant (data not shown). This finding established that the N45E stimulated transcription of P*_sspE_*-*lacZ* was dependent on σ^G^ itself. None of the other *sigG* mutations screened increased expression of P*_sspE_*-*lacZ*, as illustrated by the *sigGV44I* mutant, bearing a valine to isoleucine substitution at codon 44 (V44I) (Figure 2A and 2B).

We hypothesized that the N45 residue was a contact site for a putative inhibitor of σ^G^, which was eliminated by the N45E substitution. As a test of this idea we replaced the asparagine residue by an alanine (henceforth N45A), a substitution expected to remove any positive contribution of the wild type amino acid side chain to a presumed interaction while maintaining protein structure [34]. Unexpectedly, the N45A substitution did not increase σ^G^-directed transcription on colonies of cells growing on LB medium nor on liquid medium cultures (Figure 2A and 2B). This observation suggests that the side chain of N45 may not be essential for a direct interaction of σ^G^ with an inhibitory factor. One alternative possibility is that N45E interferes with the binding of a putative inhibitor to σ^G^.

### Activity of σ^GN45E^ during sporulation

We next studied the effect of the N45E substitution on the activity of σ^G^ during sporulation in liquid Difco sporulation medium (DSM). In this system, sporulation is induced by exhaustion of key nutrients, and its onset defined as the point at which a culture enters stationary phase. None of the *sigG* mutants that we screened in LB medium caused a Spo^−^ phenotype (data not shown), but we looked at P*_sspE_*-*lacZ* transcription during sporulation as the mutations could alter the normal activity profile of σ^G^. In wild type cells, expression of P*_sspE_*-*lacZ* was sharply induced 4 hours after the onset of stationary phase and reached maximum levels around hour 6 (Figure 2C). In keeping with the link between the activity of σ^G^ and engulfment completion, induction of P*_sspE_*-*lacZ* expression at hour 4 coincided with forespore engulfment in most cells of the population, as judged by FM4-64 staining (not shown). In N45E cells P*_sspE_*-*lacZ* expression followed a bi-modal pattern, with an early period that peaked 2 hours after the onset of stationary phase and a second, starting at hour 4, superimposable to the window of σ^G^ activity seen for wild type cells (Figure 2C). The activity profile of σ^G^ and σ^GN45E^ paralleled the accumulation of the proteins, as assessed by immunobloting with an anti-σ^G^ antibody [17]. Both σ^G^ and σ^GN45E^ accumulated to maximum levels at hour 4 of sporulation in consonance with the main period of P*_sspE_*-*lacZ* expression, following engulfment completion (Figure S2C). However, σ^GN45E^ begun to accumulate earlier than the wild type protein, soon after the onset of stationary phase in DSM, which correlates with the first period of P*_sspE_*-*lacZ* expression in the N45E mutant (Figure S2C).

Only the second period of P*_sspE_*-*lacZ* expression was seen for the V44I and the N45A mutants (Figure 2C), consistent with the observation that these mutations did not enhance expression of the reporter fusion in our initial screen (see above). Also consistent with the conclusion of our initial screen that the increased expression of the P*_sspE_*-*lacZ* reporter was not indirectly caused by titration of a negative regulator of σ^F^ (see above), the N45E mutation did not increase expression of a *lacZ* fusion to the promoter for a gene, *spoIIQ*, controlled by σ^F^ (*spoIIQ-lacZ*, [13]) (Text S1 and Figure S2A). In addition, the first period of σ^GN45E^ activity was still observed independently of *sigF*, coding for σ^F^, which normally drives transcription of *sigG* in the forespore (Figure 2F; see also below). While the *sspE* promoter can also be utilized by σ^F^ [8], [27], it is clear that the first period of σ^GN45E^ activity is σ^F^-independent. This first period also occurred in cells with deletion mutations of the *spoIIIJ* (Figure 2E) and *spoIIIA* loci (not shown), which are required for σ^G^ activity following engulffment completion. In fact, the first peak of σ^GN45E^ activity was seen even in cells of a *spo0A* deletion mutant ([35], [36], and references therein), which codes for the master regulatory protein governing entry into sporulation and without which the asymmetric division that produces the forespore compartment does not takes place [37](Figure 2F).

Altogether, these results show that the effect of the N45E substitution on P*_sspE_*-*lacZ* transcription during stationary phase in sporulation medium was dependent on and mediated by σ^G^. The results also show that the second peak of σ^GN45E^ activity remained dependent on the normal control mechanisms that govern σ^G^ production and activation during sporulation.

### σ^GN45E^ is activated in a population of stationary phase cells

**Figure 3:**
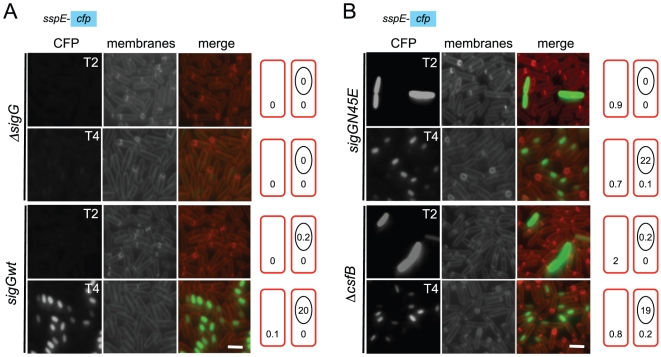
Localization of σ^G^ activity. The activity of σ^G^ was monitored by fluorescence microscopy 2 (T2) and 4 (T4) hours after the onset of stationary phase in sporulation medium (DSM), for the following panel of strains (A and B), all of which carrying a fusion of the σ^G^-controlled P*_sspE_* promoter to *cfp* at the non-essential *yycR* locus: the wild-type, a Δ*sigG* mutant, the *sigGN45E* mutant, and the Δ*csfB* mutant. The membranes were visualized with the lypophylic membrane dye FM4-64 which is unable to reach the forespore membranes following engulfment completion, and thus serves as a reporter of the engulfment status of the forespore. Fluorescence from P*_sspE_-cfp* was false colored in green. Scale bars, 1 mm. The cartoons represent the classes of cells showing CFP accumulation at the indicated times and the numbers, the percentage of cells with CFP fluorescence in the indicated compartment.

The results described in the preceding section could be explained if the N45E mutant segregated two distinct cellular populations, one with a normal pattern of σ^G^ activity, the other activating σ^G^ independently of sporulation. To test this possibility, the activity of σ^GN45E^ was localized during stationary phase in DSM, using a P*_sspE_*-*cfp* transcriptional fusion [10]. Note that under our experimental conditions, asymmetric division was completed in most of the cell population between hours 2 and 3 after entry into stationary phase (as assayed by staining with the membrane dye FM4-64), and engulfment was completed around hour 4 (above). In agreement with previous results, expression of P*_sspE_-cfp* in wild type cells was only detected in the forespore at hour 4 after the onset of stationary phase, and only in cells in which the forespore had been engulfed by the mother cell (Figure 3 and Table 1). Note that no fluorescence was detected in cells of a *sigG* deletion mutant (Figure 3), confirming that the detected expression of the fluorescent reporter relied on σ^G^. In the N45E mutant, however, around 1% of the cells scored between hour 0 and 2 after the onset of stationary phase showed strong whole-cell fluorescence (Figure 3 and Table 1). These cells had no morphological signs of sporulation, i.e., they did not show asymmetric septa or engulfing membranes as assessed by FM4-64 staining. Consistent with the absence of asymmetric septation, we found that these cells did not show P*_spo0A_*-*yfp* expression (not shown) and time-lapse microscopy experiments revealed that they eventually lysed (Figure S3). A second, larger population of N45E cells consisted of organisms that resembled the wild type in that they begun to display forespore-specific *cfp* fluorescence at hour 3 (Figure 3; Table 1). These cells did not show premature, whole-cell expression of P*_sspE_-cfp*. The results show that the first period of σ^G^ activity in the N45E mutant can be accounted for by a sub-population of cells that enter stationary phase and that do not enter in sporulation.

**Table 1:** Localization of CFP expressed from the σ^G^-controlled P*_sspE_* promoter.

		% of cells displaying CFP fluorescence in:			
Genotype	Time (h)^a^	PS^b^	MC^c^	WC^d^	Total cells
*sigG^wt^*	0	0	0	0	1407
	1	0	0	0.06	1591
	2	0.19	0	0	1574
	3	1.5	0	0	1070
	4	19.5	0	0.075	1333
	5	40.9	0	0	1287
*sigG^N45E^*	0	0	0	1.3	1282
	1	0	0	0.68	1468
	2	0	0	0.93	1070
	3	2.1	0	1.1	1011
	4	22	0.09	0.65	1070
	5	50.3	0	0.5	1187
*csfB*	0	0	0	0.61	1301
	1	0	0	1.64	1465
	2	0	0	2	678
	3	0.68	0	1.02	1175
	4	18.6	0.2	0.78	1020
	5	47.2	0.19	0.56	1061
*csfBsigG^N45E^*	0	0	0	1	1429
	1	0	0	2.1	1153
	2	0	0	1.5	1293
	3	0.22	0	1	1355
	4	12.7	0	1	1207
	5	46.9	0.29	0.7	1032

a h - hours after the onset of sporulation (T0);

b PS – prespore;

c MC – mother cell;

d WC – whole cell expression prior to asymmetric division. No whole cell expression was detected in cells that had undergone asymmetric division.

### *csfB* and the *sigGN45E* allele are epistatic

We then focused our attention in the mechanism of activation of σ^GN45E^ in post-exponential phase cells. We considered the possibility that the N45E substitution made σ^G^ less responsive to the SpoIIAB anti-σ^G^ factor, which binds to and contributes to the negative regulation of σ^G^ in non-sporulating cells [8], [12], [24]. However, we found the activity of σ^GN45E^ to remain sensitive to SpoIIAB in vivo (Text S1 and Figure S2B). While the possibility that the N45E substitution made σ^G^ refractory to SpoIIAB seemed discarded, the profile of σ^GN45E^ activity, in particular the first period of activity detected in stationary phase DSM cultures, was reminiscent of the effect reported for a mutation in *csfB*, which codes for the CsfB anti-σ^G^ factor [8], [20], [27]. For this reason, we examined the contribution of a *csfB* deletion mutation to the effect of the *sigGN45E* allele on σ^G^-directed gene expression. On LB medium supplemented with X-Gal, the double mutant exhibited levels of β-galactosidase activity similar to the single *sigGN45E* or *csfB* mutants (Figure 2A and 2B). In DSM the double mutant showed the bi-modal temporal pattern of P*_sspE_*-*lacZ* expression seen for the *csfB* or *sigGN45E* single mutants, but with β-galactosidase levels during the first period of expression higher than those of the *sigGN45E* mutant (Figure 2D). There was no detectable effect of the mutations alone or in combination, on the second period of P*_sspE_*-*lacZ* activity (Figure 2D). When examined by fluorescence microscopy, the *sigGN45E*/*csfB* double mutant resembled the single mutants: about 1% of the cells displayed early whole-cell fluorescence (between hours 0 and 2 of sporulation) whereas most of the population showed CFP fluorescence in the forespore following engulfment completion (Figure 3 and Table 1). Presumably, the fraction of pre-divisional cells with a strong whole-cell CFP signal corresponds to the β-galactosidase producing cells during the first hours of sporulation (Figure 2C–2F). In conclusion, *sigGN45E* cells phenocopied the *csfB* mutant and the *sigGN45E*/*csfB* double mutant did not differ significantly from either single mutant. These findings suggest that the *csfB* and *sigGN45E* alleles exert their effect on σ^G^ by acting on the same pathway.

### The N45E substitution reduces binding of CsfB to σ^G^

**Figure 4:**
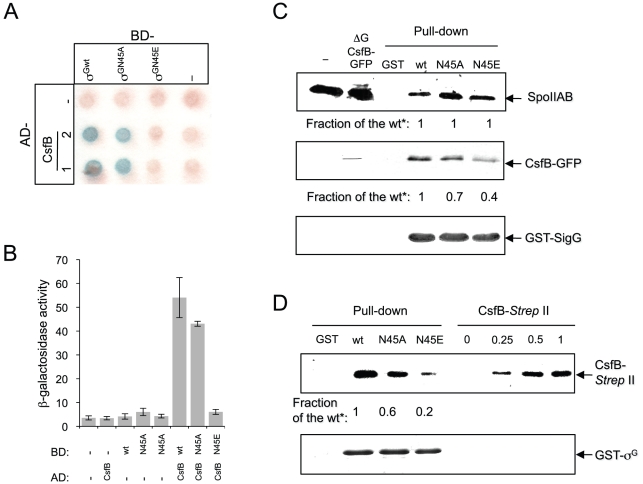
Interactions of SpoIIAB and CsfB with σ^G^, σ^GN45A^, and σ^GN45E^. Panels A and B: colony lift (A) and quantitative assay on liquid medium (B) for the detection of β-galactosidase activity in yeast strains expressing fusions of CsfB to the GAL4 activation domain (AD; two colonies are shown) and fusions of σ^G^, σ^GN45A^ or σ^GN45E^ to the GAL4 binding domain (BD), as indicated. Assays in which the BD and AD were expressed from empty vectors were used as negative controls (“−”). β-galactosidase activity was detected with X-Gal after a reaction time of 30 min (A), or with the ONPG substrate (B) and expressed in Miller units. The data represented in panel B are the average of three independent experiments. Panel C shows the results of pull-down with GST (lane GST), GST-σ^G^, GST-σ^GN45A^, or GST-σ^GN45E^ fusion proteins (as indicated above the panel) immobilized on glutathione agarose beads. Cell extracts from a *B. subtilis* strain producing a CsfB-GFP fusion were prepared from DSM cultures 2 hours into stationary phase, and incubated with the various beads preparations. Bound proteins were visualized, following elution, by immunoblotting with anti-SpoIIAB and anti-GFP antibodies. The extract used in the assay (ΔG CsfB-GFP) as well as an extract prepared from the wild type MB24 strain (control for the GFP antibody) at hour 2 of sporulation in DSM, were also directly loaded on the same gel. Panel D: GST pull-down assays with purified CsfB-*Strep* II tag. GST and the various GST-σ^G^ fusions (wt, N45A, and N45E, as indicated) were bound to glutathione beads and incubated with purified CsfB-*Strep* II (100 nM). Bound proteins were detected, following elution, with an anti-*Strep* II tag antibody. In the lanes denoted “CsfB-*Strep* II” the indicated amounts of purified protein (in ng) were analyzed by immunoblot as a control for the intensity of the signal in the pull-down experiments. In panels C and D, “Fraction of the wt*” refers to the binding ratio between of GST-σ^G^, GST-σ^GN45A^, or GST-σ^GN45E^ to SpoIIAB or to CsfB (C), or CsfB-*strep* II tag (D). The numbers were determined through densitometric analysis of the blots, and are averages of three independent experiments.

The idea that both *csfB* and the *sigGN45E* allele act on the same pathway suggested to us that the N45E substitution could interfere with binding of CsfB to the mutant form of σ^G^. In earlier work, CsfB and σ^G^ were found to directly interact in a yeast two-hybrid assay, and the first 71 residues of σ^G^ to be required for the CsfB-dependent inhibition of σ^G^ in vivo [20]. We used a similar approach to investigate whether σ^GN45E^ was less efficiently bound by CsfB. σ^G^, σ^GN45A^, σ^GN45E^ or CsfB were translationally fused to the C-terminus of the Gal4 DNA binding (BD) and activation domains (AD), and the various fusion proteins expressed in different combinations in yeast cells and checked for their ability to interact in vivo, as assessed by the expression of a *lacZ* gene preceded by a Gal4-responsive element. As shown in Figure 4A and 4B), CsfB interacts efficiently with σ^G^ and only slightly less well with σ^GN45A^. In contrast, CsfB interacts only weakly with σ^GN45E^.

We then used affinity chromatography to further investigate the interaction between CsfB and the different forms of σ^G^. Whole cell extracts were prepared from cultures of a *B. subtilis* strain producing a functional CsfB-GFP fusion, 2 hours after the onset of sporulation, when σ^F^ is active and CsfB is known to accumulate [29]. The extracts were incubated with GST-σ^Gwt^, GST-σ^GN45A^, GST-σ^GN45E^ or GST alone bound to glutathione agarose beads. Bound proteins were eluted and identified by immnunoblot with an anti-GFP antibody (see Materials and Methods). These experiments showed that CsfB was pulled down efficiently by immobilized GST- σ^Gwt^ but not by GST itself (Figure 4C). GST-σ^GN45A^ pulled down CsfB-GFP less efficiently that the wild type (the efficiency was 0.7× of the wild type) but importantly, for σ^GN45E^ the efficiency of the pull down was about 0.4× of the wild type (Figure 4C; note that the numbers in the panel represent averages for three independent experiments). We also note that in these assays SpoIIAB was pulled down by all forms of GST-σ^G^ with similar efficiency (Figure 4C), suggesting that the N45A or N45E substitutions do not significantly affect binding of SpoIIAB to σ^G^, and in line with the results of the in vivo activity experiments in which σ^GN45E^ was still susceptible to SpoIIAB (see above; Figure S2A). To discard the possibility that the reduced retention of CsfB by σ^GN45E^ was caused by increased binding of a competing protein present in the *B. subtilis* extracts, the assay was repeated using a CsfB-*Strep* II-tagged protein overproduced and purified from *E. coli*. CsfB-*Strep* II was soluble when overproduced in a minimal medium only in the presence of Zn^2+^, or in LB, which contains high levels of Zn^2+^ (Text S1 and Figure S4). The CsfB-*Strep* II protein purified from LB medium had bound Zn^2+^ (metal to protein ratio of 1∶1), as determined by atomic absorption spectroscopy. We incubated purified CsfB with GST or the various GST-σ^G^ forms immobilized on glutathione beads. After washing, CsfB was detected in the eluates by immunoblot with an anti-*Strep* II tag antibody. The CsfB protein was retained by GST-σ^G^ and by GST-σ^GN45A^ (∼0.6× the efficiency of the wild type), and to a lower level (∼0.2× of the wild type) by GST-σ^GN45E^ (Figure 4D). In these experiments, the signal in the pull down could be matched to that of a dilution of purified CsfB-*Strep* II (Figure 4D). Although the differences between σ^G^/σ^GN45A^ and σ^GN45E^ were more pronounced in the yeast two-hybrid experiments, both this assay and the pull-downs are in general agreement. Together, the results show that N45E is the substitution with the greatest impact on binding of CsfB to σ^G^.

### A negative feedback loop that limits the ectopic activation of σ^G^

**Figure 5:**
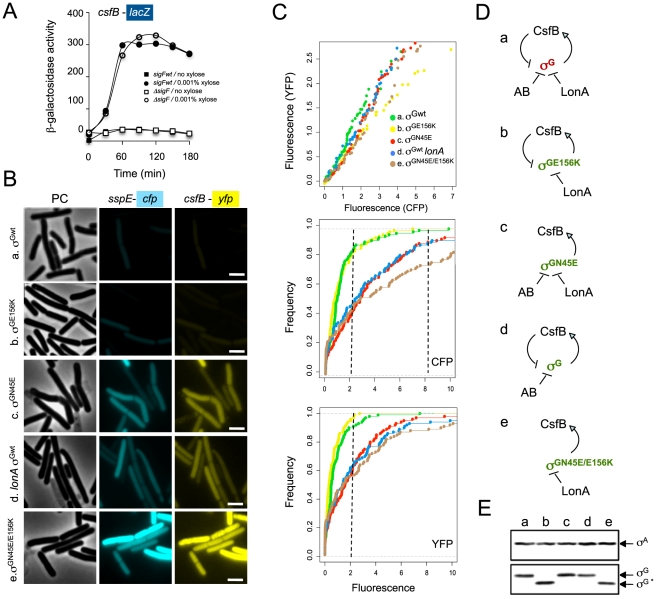
σ^G^ drives expression of *csfB* under non-sporulation conditions. Panel A: Strains carrying a P*_csfB_*-*lacZ* fusion, a xylose-inducible P*_xylA_*-*sigG* construct and the wild type *sigF* gene or a Δ*sigF* deletion, were grown to mid-log phase in LB medium and induced (circles) or not (squares) with 0.001% xylose. Samples were collected at the indicated time intervals and assayed for β-galactosidase activity (shown in Miller units). Panel B, σ^G^ activity and *csfB* expression was monitored in the same cells, by fluorescence microscopy, at the onset of stationary phase in LB. The strains used carry P*_sspE_*-*cfp* and P*_csfB_*-*yfp* reporter fusions, a deletion of the *sigG* gene, and a copy of the wild type *sigG* gene (a), *sigGN45E* (b), *sigGE156K* (c), wild type *sigG* in a *lonA* deletion mutant (d), or *sigGN45E*/*E156K* (e) at the *amyE* locus under the control of P*_xylA_*. The strains were grown in the presence of 0.001% xylose. Scale bar, 2 µm. Panel C: quantitative analysis of CFP and YFP expression for the strains (a to e) examined in B. The top plot shows the correlation between the YFP (*csfB-yfp*) and CFP (P*_sspE_-cfp*) signals. The middle and bottom graphs show a cumulative frequency distribution of the YFP and CFP signals across the population. Fluorescence intensity is shown in arbitrary units; 100 cells were scored. Panel D shows the schematic representation of the regulatory interactions in the experimental situations shown in panels A and B (inactive and active σ^G^ in brown and green, respectively). Panel E: immunoblot analysis of σ^G^ accumulation in the strains (a-e) defined in panel B. Strains were grown in LB medium and samples taken at the onset of the stationary phase for microscopy or immunoblot analysis. “σ^G^” represents the position of the wild type protein or σ^GN45E^. The asterisk denotes σ^GE156K^ or σ^GN45E/E156K^, both of which, because of the E156K substitution, migrate slightly faster than the wild type or σ^GN45E^ proteins [24].

*csfB* was first identified as a gene under the control of σ^F^, and hence transcribed in the forespore soon after asymmetric septation [29]. Yet, in our hands, the main effect of a *csfB* deletion on the activity of σ^G^ activity was manifested in predivisional cells, i.e., before the activation of σ^F^ (Figure 3). Previously, Chary *et al.*, (2007) have speculated that there is a basal level of σ^F^-directed transcription during vegetative growth. However, our results suggest that the increased activity of σ^GN45E^, which as we show is at least partially resistant to CsfB, was dependent solely on σ^G^ (see above). Therefore, and although the expression of *csfB* in the forespore is not thought to be controlled by σ^G^ [38], it seemed plausible that transcription of *csfB* in pre-divisional cells could be at least in part, controlled by σ^G^. As a first test to this idea, we investigated whether expression of *csfB* and the activity of σ^GN45E^ co-localized. We first replaced the wild type *csfB* allele by a *csfB-yfp* fusion. This *csfB-yfp* fusion was subsequently transferred to strains carrying either the wild type or the N45E alleles of *sigG* and in addition, the σ^G^ reporter P*_sspE_-cfp* [10]. In the wt strain grown in DSM, organisms began to display forespore-specific *yfp* fluorescence between hour 1 and 2 after the onset of stationary phase (Table 2), consistent with the timing of septation and the activation of σ^F^. No fluorescing organisms were observed before hour 1, or in cells of a Δ*sigF* mutant, as expected for a σ^F^-controlled gene (Table 2; data not shown). Conversely, *cfp* fluorescence was only observed in the forespore 2 hours after the onset of sporulation, as expected for a σ^G^-controlled gene and demonstrating the functionality of the *csfB-yfp* fusion. In the N45E mutant, between 1% to 4% of the bacteria displayed both whole-cell YFP and CFP fluorescence during the first hours of stationary phase in DSM (Table 2). Around hour 2, the first cells showing forespore-specific expression of *csfB-yfp* were detected followed, around hour 3, by cells with engulfed forespores showing P*_sspE_*-*cfp* expression. From this analysis, it is clear that the whole-cell expression of *csfB-yfp* early in stationary phase is confined to cells that also show activity of σ^GN45E^, suggesting that *csfB-yfp* was transcribed under the direction of σ^G^. As a further test to the possibility that σ^G^ controlled transcription of *csfB*, we made use of the *sigG* inactive allele described above in which the N45E mutation was combined with the F91A and Y94A “promoter-melting” mutations (Figure S1). In cells of the triple *sigG* mutant, no whole-cell YFP or CFP fluorescence was detected. In addition, and as expected, cells of the triple mutant did not display forespore-specific CFP fluorescence (which is σ^G^-dependent) but showed forespore-specific YFP fluorescence (which is σ^F^-dependent) (Table 2). These results strongly suggest that the expression of *csfB* in pre-divisional cells is σ^G^-dependent, a conclusion reinforced by the observation that deletion of *sigF* in a N45E background abolished both the forespore-specific YFP and CFP fluorescence (σ^F^- and σ^G^- dependent) while maintaining the early whole-cell expression of *yfp* and *cfp* (Table 2). Lastly, as a more direct test for the ability of σ^G^ to control the expression of *csfB*, we monitored the expression of a P*_csfB_-lacZ* fusion upon artificial induction of σ^G^ production from P*_xylA_* in vegetatively growing cells. The results in Figure 5A show that addition of xylose resulted in the induction of *csfB-lacZ* expression, even in the presence of a *sigF* deletion mutation, consistent with the view that σ^G^ can also drive expression of *csfB*, and with the similarity of the −10 and −35 promoter elements recognized by σ^F^ and σ^G^ [39]–[41].

**Table 2:** Localization of *csfB-yfp* and P*_sspE_*-*cfp* expression.

		% of cells displaying CsfB-YFP/P_sspE_-CFP fluorescence in:		
Genotype	Time (h)^a^	PS^b^	WC^c^	Total cells
*sigG^wt^*	0	0/0	0/0	1356
	1	0.9/0	0/0	778
	2	2.1/0.2	0/0	1717
	3	27.3/4.3	0/0	1469
*sigG^N45E^*	0	0/0	3.7/2.8	646
	1	0/0	1.5/1.0	397
	2	2.8/0.3	0.8/0.5	769
	3	30.1/1.3	0.9/0.3	626
*sigG^F91Y94^*	0	0/0	0/0	1014
	1	0.4/0	0/0	774
	2	5.5/0	0/0	1299
	3	25.0/0	0/0	629
*sigG^N45EF91Y94^*	0	0/0	0/0	1978
	1	0.1/0	0/0	1491
	2	4.9/0	0/0	1708
	3	37.0/0	0/0	404
*sigFsigG^N45E^*	0	0/0	2.5/1.6	947
	1	0/0	1.2/1.0	1317
	2	0/0	0.5/0.2	864
	3	0/0	0.2/0	925

a h - hours after the onset of sporulation (T0);

b PS – prespore;

c WC – whole cell expression prior to asymmetric division. No whole cell expression was detected in cells that had undergone asymmetric division.

Taken as a whole, the results suggest that the capacity of σ^G^ to drive production of CsfB in pre-divisional cells may be part of a mechanism to limit the ectopic activation of σ^G^ should any condition promote its activation.

### Genetic lesions that relax the regulation of σ^G^ also result in CsfB production

If production of CsfB is part of a regulatory circuit that self-restrains the activity of σ^G^, then mutations in other factors known to negatively regulate σ^G^ should also induce expression of *csfB*, and the extent of the effect across the population should reflect the contribution of the affected regulator to the regulation of σ^G^. Two such factors are known, the LonA protease and the SpoIIAB anti-sigma factor, which act independently to negatively regulate the activity of σ^G^, mainly under non-sporulation conditions [21], [22]. To determine the relative impact of mutations known to affect the regulation of σ^G^ on its activity across the population, and whether those mutations also increased the expression of *csfB*, we used fluorescence microscopy to simultaneously quantify the expression of P*_sspE_-cfp* and *csfB-yfp* at the onset of stationary phase in LB, in a panel of strains carrying P*_xylA_* fusions to wild type *sigG*, *sigGN45E*, *sigGE156K* (coding for a form of σ^G^ refractory to SpoIIAB; [24]), *sigGN45E/E156K* or a P*_xylA_*-*sigG* ^wt^ construct in combination with a *lonA* deletion mutation (Figure 5B). The growth medium was supplemented with 0.001% xylose, as in preliminary experiments (Text S1 and Figure S5) this was the highest concentration at which wild type σ^G^ could be induced without causing significant cell lysis. In control experiments, no fluorescence could be detected in strains lacking either of the P*_sspE_-cfp* and *csfB-yfp* fusions (not shown).

The results in Figure 5C (top graph) show a clear correlation between the YFP and CFP signals for all strains tested. Cells that produce CFP also produce YFP, and an increase in the expression of one reporter is accompanied by an increase in the expression of the other (Figure 5C, top), highlighting the link between the activity of σ^G^ and the production of its negative regulator, CsfB. The middle and lower graphs of Figure 5C are cumulative frequency distributions of the CFP and YFP signals for the various strains. For the N45E, *lonA*, and N45E/E156K strains, about 50% and 40% of the population shows CFP and YFP signals, respectively, above 2 arbitrary units. In contrast, only 10% of the wt or E156K populations show CFP or YFP signal intensities above this value (Figure 5C). Induction of σ^GN45E/E156K^ increased the number of cells with high CFP fluorescence (above 8 arbitrary units) to 20% of the population, as compared to 10% for the strains bearing the single N45E, E156K or *lonA* mutations. This observation is in agreement with the idea (see above) that σ^GN45E^ is still sensitive to SpoIIAB. Smaller differences in the YFP signal distribution were seen between the double N45E/E156K mutant and the single N45E, E156K and *lonA* mutants, possibly reflecting reduced YFP stability. While CsfB, mainly, and LonA emerge as the principal regulators of σ^G^ activity during entry into the stationary phase of growth, SpoIIAB *per se* seems to have only a minor role (Figure 5C). Importantly, we were unable to combine the *sigGN45E*/*E156K* allele with a *lonA* deletion, highlighting the convergent action of CsfB, SpoIIAB and LonA in the negative regulation of σ^G^, and suggesting that these are likely to be the main, if not the only, negative regulators of σ^G^ at play. The results also unravel a negative σ^G^ autoregulatory loop (Figure 5D), in which by commanding the expression of *csfB*, the fraction of cells with ectopic activity of σ^G^ is curtailed.

Since production of σ^G^ in the strains above was driven from the P*_xylA_* promoter, we expected the various forms of σ^G^ to accumulate to similar levels, independently of the number of cells showing σ^G^ activity. This was verified by immunobloting analysis with an anti-σ^G^ antibody, for the N45E, E156K, N45E/E156K and wild type σ^G^ in the *lonA* background (Figure 5E).

### CsfB discriminates between σ^F^ and σ^G^ via one single amino acid

Because changing N45 of σ^G^ for the residue found at the equivalent position in σ^F^, E39, makes σ^G^ less efficiently bound by CsfB, and since CsfB does not bind to σ^F^ [20], we reasoned that perhaps this position was essential for the discrimination by CsfB between the two forespore-specific sigma factors in vivo. This inference was strengthened by the observation that N45 is invariant in σ^G^ orthologues, whereas E39 is highly conserved among σ^F^ proteins (Figure 1C).

**Figure 6:**
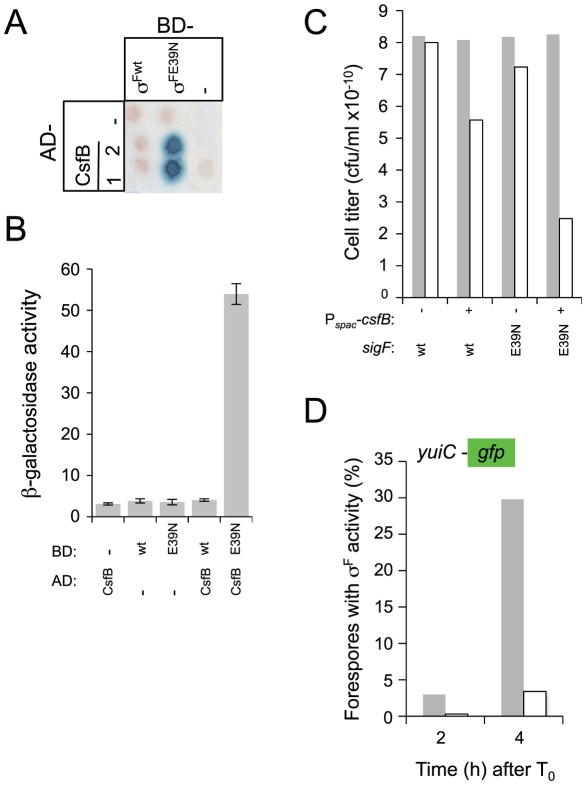
Interactions of CsfB with σ^F^ and σ^FE39N^. Panels A and B show the results of a colony lift (A) or a quantitative assay on liquid medium (B) for the detection of *lacZ* transcription in yeast strains expressing fusions of CsfB to the activation domain (AD; two colonies are shown) and fusions of σ^F^ or σ^FE39N^ to the binding domain (BD) of GAL4. Negative controls (“−”) included BD and AD expressed from empty vectors. β-galactosidase activity was detected as described in the legend for Figure 4. The results in B are the average of three independent experiments. Panel C shows the efficiency of sporulation of strains bearing a wild type copy of the *sigF* operon, or a copy with the *sigFE39N* allele, at *amyE*. In addition, the strains carry the *csfB* gene inserted at the *thrC* locus under the control of the IPTG inducible P*_spac_* promoter (Table S1). The two strains (P*_spac_*-*csfB spoIIAABC wt* and P*_spac_*-*csfB spoIIAABCE39N*; NB: the third cistron of the *spoIIA* operon, also called *sigF*, codes for σ^F^) were grown in DSM in the presence (+) or in the absence (−) of IPTG and the titer of heat resistant spores measured 18 hours after the onset of stationary phase (gray bars, viable cells; white bars, heat resistant cells). Panel D, shows the quantitative analysis of GFP expression in strains similar to those described for panel C, except that they additionally carry a fusion of the σ^F^-controlled *yuiC* promoter to *gfp*. The two strains (P*_spac_*-*csfB sigF^wt^* P*_yuiC_-gfp*, gray bars; P*_spac_*-*csfB sigFE39N* P*_yuiC_-gfp*, white bars) were grown in sporulation medium (DSM) in the presence of IPTG and samples taken at the indicated times (in hours) after the onset of stationary phase for scoring the number of cells showing GFP expression by fluorescence microscopy.

Therefore, we decided to investigate whether E39 was important for the resistance of σ^F^ to CsfB, and for the regulation of its activity in vivo. We conducted GAL4-based yeast two-hybrid experiments to test the interaction between wild type σ^F^ and a mutant form of the protein with the E residue at position 39 replaced by an N (E39N; Figure 1B). In agreement with the results of an earlier study [20], CsfB did not interact with σ^F^ in our assay (Figure 6A and 6B). In contrast, CsfB interacted efficiently with σ^FE39N^ (Figure 6A and 6B). Thus, the E39N substitution is sufficient to allow binding of CsfB to σ^F^.

We next wanted to test whether the presence of an N at position 39 of σ^F^, expected to make it susceptible to CsfB, would affect spore development. We found the E39N substitution to cause a 5-fold decrease in the efficiency of sporulation (data not shown). Chary *et al.* found that when *csfB* is expressed from the IPTG-inducible P*_spac_*_(Hy)_ promoter prior to the activation of σ^F^, spore formation was severely reduced [27]. However, in this strain the activity of σ^F^ was not impaired and spore formation was blocked in the developmental pathway just after engulfment completion [27]. We used a similar assay to test for the effect of the E39N mutation on the activity of σ^F^. We transferred the IPTG-inducible P*_spac_*_(Hy)_-*csfB* fusion to strains mutant for *sigF* and with a second copy of the entire *sigF* operon (with either the wild type *sigF* cistron or *sigFE39N*) integrated at the *amyE* locus under the control of its native promoter. In the strain carrying the wild type allele of *sigF* grown in the presence of IPTG to induce *csfB* expression prior to the activation of σ^F^, spore formation showed the reported 10^3^-fold reduction relative to cultures without IPTG (Figure 6C) confirming the results of Chary *et al.* (2007). Strikingly, induction of *csfB* expression in the strain carrying the *sigFE39N* allele of *sigF* reduced spore formation 10^6^-fold compared to the uninduced cultures (Figure 6C). To investigate whether the more drastic sporulation defect observed in the strain carrying the E39N allele was due to impaired σ^F^ activity, we used a fusion of the σ^F^-dependent *yuiC* promoter to *gfp* [42]. GFP fluorescence was monitored by microscopy during sporulation in the strains with IPTG inducible expression of *csfB* and bearing either the wild type or *sigGE39N* alleles. We found that the induction of *csfB* reduced the activity of σ^FE39N^ to 10% of the levels observed when CsfB was produced prior to asymmetric division in the presence of wild type σ^F^ (Figure 6D). Thus, CsfB can interfere strongly with the activity of σ^FE39N^ in vivo.

## Discussion

### CsfB and the feedback inhibition of σ^G^

The production of transcription factors often leads to the activation of gene expression during cell differentiation and development. In some instances, positive auto-regulation of the transcription factor drives gene expression in the differentiating cell down a specific developmental path. However, the power of these positive feedback loops raises the potential for inappropriate expression of the transcription factor in the wrong cell or at the wrong time. Therefore, the expression of autoregulatory transcriptional activators must be tightly controlled. We show here that CsfB has a function in preventing the activation of the forespore-specific, auto-regulatory σ^G^ factor, in stationary phase cells, in either a medium that does not support sporulation or in a sporulation medium, prior to the asymmetric division that initiates the program of compartment-specific gene expression that leads to differentiation of the spore.

This role of CsfB was uncovered because the N45E substitution in σ^G^, which reduces binding by CsfB, also results in activation of the sigma factor in a fraction of stationary phase cells. We show that CsfB is also produced, under σ^G^ control, in the same stationary phase cells where σ^GN45E^ becomes active. Hence, a negative feedback loop is established which, with the help of SpoIIAB and LonA, dominates the positive feedback loop involving σ^G^, and keeps its activity low. The role of LonA and SpoIIAB in the negative regulation of σ^G^ in stationary phase cells was shown before [9], [12], [17], [21], [22], but our analysis suggests that CsfB and LonA are the main regulators of σ^G^. Nevertheless, the role of SpoIIAB is evidenced when the N45E and E156K substitutions are combined, and by our inability to construct a strain additionally carrying a *lonA* deletion. The lethality of this triple mutant further suggests that CsfB, SpoIIAB and LonA may be the only negative regulators of σ^G^ at play in stationary phase cells.

Although mutations that interfere with the function of CsfB, SpoIIAB or LonA may cause strong expression of σ^G^-dependent genes in pre-divisional cells, this only occurs in a fraction of the population (Figure 5). We do not presently know whether the cells which show σ^G^ activity are somehow different from the rest of the population at some fundamental level, or whether σ^G^ activity arises because of random fluctuations in the levels of σ^G^ itself, and its negative regulators. In any case, high-level expression of even wild type σ^G^ in stationary phase cells causes cell lysis, emphasizing the importance of limiting the potential for σ^G^ activation (Figure S6). Lysis may be a consequence of high levels of σ^G^ activity [26], an indirect effect of the release of σ^F^ through titration of SpoIIAB by σ^G^ [20] or both, as induction of σ^G^ production in LB leads to lysis even in the absence of σ^F^ (data not show).

### CsfB and the activation of σ^G^

CsfB was initially proposed to be a key factor in keeping σ^G^ inactive in the forespore prior to engulfment completion [20]. However, a more consensual view of the role of CsfB is that the anti-sigma factor acts as a timing device, to help prevent σ^G^ activity prior to engulfment completion [8], [9], [27], [38]. The results of our investigation are in line with this view, as in our hands deletion of *csfB* or the N45E substitution in σ^G^ (which we show prevents binding of CsfB to σ^G^) did not bypass the genetic and morphological controls that link the activity of σ^G^ to engulfment completion. We postulate that during sporulation the negative feedback loops contributes to counter the positive feedback loop involving σ^G^ until CsfB is inactivated, or its synthesis is reduced by an unknown second regulator, or σ^G^ accumulation overwhelms that of CsfB. It is not known if CsfB is inactivated in the forespore following engulfment completion, but the anti-sigma factor seems to rapidly disappear from the forespore once σ^G^ becomes active (our unpublished results). It is also likely that an additional factor prevents expression of *csfB* in the engulfed forespore. For example, σ^G^ drives production of SpoVT a forespore-specific transcription factor, which represses at least 27 σ^G^-dependent transcriptional units [41], [43]. SpoVT has a C-terminal GAF (cGMP-specific and cGMP-stimulated phosphodiesterases, *Anabaena* adenylate cyclases, and *Escherichia coli* FhlA)-like domain, which is essential to modulate the DNA-binding activity of the N-terminal domain, and may respond to nucleotides or other small molecules [44]. The accumulation of nucleotides in the engulfed forespore in turn, may be essential for the activity of σ^G^ and may depend on the action of the SpoIIIA-Q channel [9].

### Binding of CsfB to σ^G^

Two observations are consistent with the interpretation that the N45E substitution interferes with binding of CsfB to σ^G^. First, wild type σ^G^ could pull down CsfB from extracts of *B. subtilis* in sporulation medium, but σ^GN45E^ did so less efficiently, a difference that was amplified when purified CsfB was used (Figure 4; see also below). Second, CsfB interacted with wild type σ^G^ but not with σ^G^ N45E in a yeast two-hybrid system (Figure 4). CsfB was purified from *E. coli* cells as a C-terminal fusion to the *Strep* II tag because *in vivo* a CsfB-GFP fusion was fully functional (this work). The CsfB-*Strep* II protein had Zn^2+^ bound with a stoichiometry of 1∶1. The Zn^2+^ was released by oxidation of the protein with H_2_O_2_, suggesting the involvement of the conserved Cys residues in CsfB in its coordination (see Figure S4). Recently, a MalE-CsfB fusion protein was purified from sporulating cells of *B. subtilis* with Zn^2+^ bound with a stoichiometry of 0.5 mol/mol [28]. Together with genetic data, this suggested that CsfB could act as a dimer (or higher order multimer) and possibly alternate between an active and an inactive state [28]. Importantly, the activity of σ^G^ was efficiently inhibited in *E. coli* cells, when co-produced with CsfB [28], and the CsfB-*Strep* II protein purified from *E. coli* cells clearly discriminated σ^G^ and σ^GN45E^ in our pull-down assays (Figure 4E).

The N45 residue in σ^G^ may contribute to the interaction with CsfB. If so, however, this contact does not seem to be essential because the N45A substitution did not result in increased activity of σ^G^ in vivo, and caused only a small reduction in the ability of σ^G^ to interact with CsfB in yeast two-hybrid and pull-down assays (Figure 4). Additional mutagenesis studies may illuminate if and how the N45 residue contributes to the interaction with CsfB, and how the N45E substitution interferes with the interaction. CsfB is likely to contact σ^G^ at other positions, and these other contact sites are likely to be present in σ^F^ as well. First, because no other single mutation was found within the first 150 residues of σ^G^ that would affect its activity in vivo and second, because while incapable of binding to wild type σ^F^ ([20]; this work), CsfB bound efficiently to σ^FE39N^ (see also below). The location of the N45 residue within region 2.2 of σ^G^ and its role in permitting binding by CsfB, is also consistent with previous work in which a σ^F/G^ chimeric protein allowed the target for CsfB to be mapped within the first 77 residues of σ^G^ [20]. The N45 residue is invariant among σ^G^ orthologues of *Bacillus* species and related organisms but less conserved among the σ^G^ proteins of the Clostridia (Figure 1C). These observations highlight the importance of N45 (and homologous residues) in a sub-group of sporeformers including *B. subtilis* and related organisms, in which σ^G^ is regulated by the anti-sigma factor CsfB.

### The N45E substitution may also affect a contact with the β′subunit of core RNA polymerase

The observation that the N45E substitution reduces binding of CsfB to σ^G^ provides a plausible explanation for the increased activity of σ^GN45E^ in vivo. However, we cannot at present discard the possibility that the N45E alteration, which affects a residue positioned within conserved region 2.2, also increases binding of σ^G^ to core RNA polymerase. The position homologous to N45 is often occupied by an acidic residue in proteins of the σ^70^ family of sigma factors (the σ^G^ orthologues of *Bacillus* species and related organisms being a conspicuous exception), and in the crystal structure of the σ^70^-containing RNA polymerase holoenzyme from *Thermus aquaticus* [45], E189 (homologous to N45 in the σ^G^ protein of *B. subtilis*) is involved, with other neighboring residues, in a direct contact with residue K159 in the β′subunit (Text S1 and Figure S6). An asparagine residue, as is found in σ^G^, could also contribute to the interaction with β′ at this site. However, an acidic residue would most likely make a stronger, electrostatic, contribution to the interaction. This in turn suggests that the N45E substitution could also enhance the activity of σ^G^ by favoring its interaction with the β′subunit of core RNA polymerase. If so, then the regulation of σ^G^ activity in vivo could involve competition between CsfB and β′ for binding to σ^G^. In any event, the possible contact involving N45 and β′ is in line with the view that one mechanism by which anti-sigma factors function is by occluding sigma-core binding interfaces [46], [47]. Two of the mutations known to impair binding of SpoIIAB to σ^F^ map within region 2.2, and mark residues that are conserved in σ^G^ ([24], [48]; see also Figure 1B). This suggests that SpoIIAB and CsfB may use partially overlapping interfaces in binding to σ^G^ and may explain the competition between the two anti-sigma factors for binding to σ^G^ under certain conditions [20], [28], [48]. However, σ^GN45E^ was still bound by SpoIIAB and was still susceptible to SpoIIAB in vivo. Therefore binding of SpoIIAB to σ^G^ does not seem to require the N45 residue.

### Discrimination between σ^F^ and σ^G^

The strict conservancy of N45 among σ^G^ proteins of other *Bacillus* species and related organisms, together with its nearly absolute exclusion from orthologues of σ^F^, suggests an important, conserved role for this residue. CsfB does not seem to negatively modulate the activity of σ^F^, consistent with its inability to bind to this σ factor [20], [27], [28], [38]; this work). Because the E39N substitution is sufficient to allow binding of CsfB to σ^F^, the E39/N45 position in the σ^F^/σ^G^ families of proteins seems critical for the discrimination by the CsfB anti-sigma factor. Perhaps strengthening this idea, the only exception to the rule that an N is excluded from the critical position in σ^F^ is *B. clausii* (Figure 1C), but in this organism no *csfB* orthologue could be identified (not shown).

Recently, a protein related to CsfB, and termed Fin, was shown to inhibit the activity of σ^F^ and to play an important role in promoting the switch from σ^F^ to σ^G^ in the forespore [38]. It is possible that the N45/E39 residues help enforcing the specific regulation of σ^F^ by Fin and of σ^G^ by CsfB. It is not known whether σ^FE39N^ is susceptible to Fin. However, the E39N substitution did not seem to affect the activity of σ^F^ and caused only a 5 fold reduction in the efficiency of sporulation (this work), whereas deletion of *fin* increased the window of expression of σ^F^-dependent genes, and caused a 50-fold reduction in the efficiency of sporulation [38]. Perhaps then, σ^FE39N^ is still regulated by Fin. CsfB was also proposed recently, to antagonize low levels of σ^E^ resulting from inappropriate activation in the forespore, thus contributing to the confinement of its activity to the mother cell [49]. It is not yet known whether CsfB interacts with σ^E^. However, if so, and because an acidic residue (E) is found at the position equivalent to N45 in σ^G^, it follows that in the context of the σ^E^ protein binding by CsfB is likely to involve other residues.

## Materials and Methods

### Strains and general methods

The *B. subtilis* strains used in this work are congenic derivatives of the Spo^+^ strain MB24 (*trpC2 metC3*), and are listed in Table S1. The plasmids used in strain construction are described in the sections below and in Text S1. LB medium was used for growth or maintenance of *E. coli* and *B. subtilis*, and sporulation was induced by growth and exhaustion in Difco sporulation medium (DSM) [24]. The Quick Change site-directed mutagenesis system (Stratagene) was used for the generation of all site-specific mutations, which were always confirmed by sequencing.

### Mutagenesis of the *sigG* gene

We used pMS45, containing the *sigG* gene [24] and *sigG*-specific primers (all primers are listed in Table S2) to convert the residues highlighted in Figure 1B (orange and red circles) into the aminoacid found in σ^F^. The various mutations were then transferred to the *sigG* locus by congression as it has been observed that certain mutations cause a greater increase in the activity of σ^G^ when *sigG* is inserted at an heterologous locus such as *amyE* ([27]; our unpublished observations). For congression, pMS45 and its derivatives carrying the different *sigG* mutations, together with chromosomal DNA from AH6566 (Δ*sigG* Δ*yycR*:: P*_sspE_*-*cfp* Δ*sspE*::P*_sspE_*-*lacZ*), was used to co-transform strain AH2452 (Δ*sigG* Δ*sspE*::P*_sspE_*-*lacZ*) with selection to Cm^R^. Spo^+^ congressants appeared at a frequency of about 3%. One congressant for each *sigG* allele for which the presence of the desired mutation was confirmed by PCR and sequencing was kept for further study. Mutants that showed increased b-galactosidase production from the P*_sspE_-lacZ* reporter fusion were identified on LB plates containing 5-bromo-4-chloro-3-indolyl-b-D-galactopyranoside (X-Gal).

### Promoter and reporter gene fusions

The construction of fusions of the xylose-inducible *xylA* promoter to different *sigG* alleles, and of *csfB*-*gfp*, -*yfp* and *lacZ* fusions is described in detail in Text S1, accompanying this article.

### Construction of the *sigF E39N* mutant

First, the entire *spoIIA* operon was PCR amplified with primers sigF219D and sigF2032R (Table S2 lists all primers used in this study), the 1813 bp product digested with *Bam*HI and *Hin*dIII and inserted between the same sites of pDG364 [50], This created pMS393. Next, primers sigFE39ND and sigFE39NR were used to substitute the glutamate codon at position 39 of the *sigF* gene in pMS393 by an asparagine codon, which resulted in pMS394.

### Yeast two-hybrid analysis

The coding regions of *sigG*, *sigF* and *csfB* were PCR amplified with primers sigG2016D and sigG2862R, sigF493D and sigF1318R, and primers csfB191D and csfB480R. The *sigG* and *sigF* PCR products were digested with *Nco*I and *Eco*RI and inserted between the same sites of pAS2-1 (Clontech) yielding plasmids pMS358 and pMS357, respectively. We used pMS358 and primers sigGN45ED and sigGN45ER to substitute the asparagine codon at position 45 of σ^G^ by a glutamate codon. This resulted in plasmid pMS360. We used pMS358 and primers sigGN45AD and sigGN45AR to substitute the asparagine codon at position 45 of σ^G^ by an alanine codon. This resulted in plasmid pMS429. We used pMS357 and primers sigFE39ND and sigFE39NR to substitute the glutamate codon at position 39 of σ^F^ by an asparagine codon. This resulted in plasmid pMS387. The *csfB* PCR product was digested with *Nco*I and *Sal*I and inserted between the same sites of pACT2 (Clontech) yielding plasmid pMS356. Mating of *Sacharomyces cerevisiae* strains and detection of β-galactosidase activity were performed as described before [51].

### Overproduction and purification of SpoIIAB

The *spoIIAB* coding region was PCR amplified with primers spoIIAB189D and spoIIAB698R. The PCR product was digested with *Bam*HI and *Xho*I and inserted between the same sites of pET30a (+) (Novagen) creating pMS111, in which the sequence for the His_6_ tag was introduced between the first and second codons of *spoIIAB*. pMS111 was introduced into competent cells of BL21 (DE3) pLysS (Novagen). Growth, induction, and lysate preparation was essentially as described [52]. The His_6_-SpoIIAB fusion protein was partially purified on His-Trap chelating columns as described by the manufacturer (Amersham Pharmacia Biotech) and used to raise a polyclonal anti-SpoIIAB antibody in rabbits (Eurogentec, Belgium).

### Overproduction and purification of CsfB

First, primers csfB191D and csfBstrepR, which include the sequence coding for the *Strep* II tag (IBA GmbH) were used to PCR amplify the coding region of *csfB*. The resulting PCR product was digested with *Nco*I and *Bam*HI and cloned between the same sites of pET16b (Novagen) to create pMS350, which was then transformed into *E. coli* strain BL21(DE3). The *E. coli* expression strain was grown to mid-log phase in LB (0.6 optical density at 600 nm), induced with 1 mM isopropyl-D-thiogalactopyranoside (IPTG), and grown for 3 h before harvesting the cells. The cell pellets were resuspended in 3 ml portions of buffer A (100 mM NaCl, 10 mM Tris pH 8.0, 10% glycerol) per 50 ml of induced culture and lysed in a French pressure cell (18,000 lb/in^2^). The lysate was centrifuged to remove cell debris. CsfB-*Strep* II tag was purified on Strep-Tactin Sepharose columns following the manufacturer instructions (IBA GmbH). The metal content of the purified protein was analyzed by atomic absorption.

### GST pull-down experiments

Primers sigG2016D and sigG2964R were used to PCR amplify the coding regions of *sigG*, *sigGN45A* and *sigGN45E* from pMS45, pJS4, and pJS2 (see above). The PCR products were digested with *Bgl*II and *Xho*I and cloned between the *Bam*HI and *Xho*I sites of pGex4T-3 (GE Healthcare) to create pMS375, pMS376, and pMS377, respectively, which bear in-frame N-terminal GST fusions to the different forms of σ^G^. Derivatives of BL21(DE3) bearing each of these plasmids or pGex4T-3 (GST-alone) were grown to mid-log phase (O.D._600_≈0.6) in LB, and induced with 1 mM IPTG for 3 h before the cells were harvested. The cell pellets were resuspended in 1 ml portions of buffer A [100 mM NaCl, 10 mM Tris-HCl (pH 8.0), 10% glycerol] per 50 ml of induced culture and lysed in a French pressure cell (18,000 lb/in^2^). The lysate was cleared by centrifugation. One milliliter of cleared lysate was bound to 50 µl of a 50% slurry of glutathione Sepharose beads (GE Healthcare) at room temperature for 30 min. The beads were washed three times in buffer B (same as A but with 200 mM NaCl).

For the SpoIIAB and CsfB interaction assays, 1 ml portions of soluble extracts prepared from cultures of *B. subtilis* AH6608 (*csfB::km* Δ*sigG* Δ*amyE::csfB-gfp*) 2 h after the onset of sporulation were incubated for 30 min at room temperature with GST or the various GST fusions proteins bound to glutathione Sepharose beads or with the beads alone. The mixtures were washed three times with buffer B (above), resuspended in a final volume of 30 µl, and subjected to SDS-PAGE and immunoblotting. Rabbit anti-GFP (A.L. Isidro and A.O. Henriques, unpublished) and anti-SpoIIAB (above) antibodies were used at dilutions of 1∶1000 and 1∶500, respectively. An anti-σ^G^ antibody, at a 1∶1000 dilution, was used to control for the level of the GST-σ^G^ fusions immobilized [24].

For the CsfB interaction assay, 100 nM of purified CsfB-*Strep* II tag was incubated for 30 min at room temperature with the glutathione Sepharose beads complexed with the GST fusion proteins or with glutathione Sepharose beads alone. The mixtures were washed three times with buffer B (above) and resuspended in a final volume of 30 µl. The samples were subjected to SDS-PAGE and immunoblotting. An anti-*Strep* II tag polyclonal antibody was used at a 1∶1000 dilution (IBA GmbH). For graphical representation of the data, the immunoblots were scanned and analyzed using the ImageJ software (http://rsbweb.nih.gov/ij).

### Accumulation of σ^G^

Immunoblot analysis was used to monitor the accumulation of σ^G^ during growth or sporulation as previously described [17]. An anti-σ^A^ antibody was used as described before [36].

### β-Galactosidadse assays

β-Galactosidadse activity was assayed with the substrate *o*-nitro-β-D-galactopyranoside (ONPG), with enzyme activity expressed in Miller units [52].

### Fluorescence microscopy

Samples (0.6 ml) of LB or DSM cultures were collected, resuspended in 0.2 ml of phosphate-buffered saline (PBS) and the membrane dye FM4-64 (Molecular Probes) added to a final concentration of 10 µg ml^−1^. Microscopy was carried out as described previously [53]. Quantitative analysis of fluorescence intensity was done using the MetaMorph software package (MDS Analytical Technologies). Data was analyzed and plotted using the “R” statistical computing and graphics software package (www.r-project.org).

## Supporting Information

Figure S1 Panel A shows the sequence alignment of the subregion 2.3 of σ^G^ from *B. subtilis* with the same region of σ^A^ from *B. subtilis* and σ^70^ from *E. coli*. The aminoacids (F91 and Y94) of σ^G^ changed to alanine are highlighted. Panel B shows the expression of a σ^G^-dependent P*_sspE_-lacZ* fusion during stationary phase in DSM in the following strains: wild type background (AH6567, Δ*yycR*::P*_sspE_*-*cfp* Δ*sspE*::P*_sspE_*-*lacZ*, closed circles), the σ^G^ ^F91AY94A^ mutant (AH6539, *sigGF91A/Y94A* Δ*yycR*::P*_sspE_*-*cfp* Δ*sspE*::P*_sspE_*-*lacZ*, open circles). Samples were collected every hour during stationary phase in DSM and assayed for β-galactosidase activity (shown in Miller Units). Panel C shows the immunoblot analysis of σ^G^ accumulation during sporulation in a wild-type background (AH6567, Δ*yycR*::P*_sspE_*-*cfp* Δ*sspE*::P*_sspE_*-*lacZ*, panel E), and in the σ^GF91A/Y94A^ mutant (AH6539, *sigGF91A/Y94A* Δ*yycR*::P*_sspE_*-*cfp* Δ*sspE*::P*_sspE_*-*lacZ*). Samples from sporulating cultures were collected at the onset of stationary phase in DSM and at hourly intervals thereafter, as indicated by the numbers above the lanes. Lanes labelled “ΔG” in all panels contain an extract prepared from a Δ*sigG* deletion mutant at hour 4, as a control for the specificity of the antibody. σ^A^ levels were monitored as a control for loading. The position of σ^G^ and σ^A^ is indicated by arrowheads.
(EPS)

Figure S2 Panel A, expression of a σ^F^-dependent P*_spoIIQ_-lacZ* fusion was monitored during sporulation in the following strains: wild type background AH3447 (Δ*spoIIQ*::P*_spoIIQ_*-*lacZ*, closed squares), and σ^GN45E^ mutant AH6610 (*sigGN45E* Δ*spoIIQ*::P*_spoIIQ_*-*lacZ*, open squares). Samples from stationary phase cells in DSM were collected every hour and assayed for β-galactosidase activity (shown in Miller Units). Panel B, SpoIIAB inhibits the activity of σ^G^ or σ^GN45E^ in vivo. *B. subtilis* strains carrying a P*_sspE_*-*lacZ* fusion as a reporter of σ^G^-activity, a P*_spac_*-*spoIIAB* fusion to allow the inducible production of SpoIIAB, and xylose-inducible fusions to *sigG* (AH2492; P*_xylA_*-*sigG*), *sigGE156K* (AH2493, P*_xylA_*-*sigGE156K*) or *sigGN45E* (AH6556, P*_xylA_*-*sigGN45E*) were grown in LB in the presence (1 mM) or in the absence of IPTG. Samples were taken at indicated times during growth and assayed for β-galactosidase production. The graph shows the percentage of the activities of σ^G^ (black bars), σ^GE156K^ (white bars) or σ^GN45E^ (gray bars) found in the absence and in the presence of IPTG. Panel C documents the accumulation of σ^G^ and σ^GN45E^ during stationary phase in DSM using an anti-σ^G^ antibody. The accumulation of σ^G^ and σ^GN45E^ was examined in a wild-type background and in a Δ*csfB* mutant. Samples were colected at the onset of stationary phase in DSM and at hourly intervals thereafter, as indicated by the numbers above the lanes. The lanes labeled “ΔG” in all panels contain an extract from a *sigG* deletion prepared at hour 4, as a control for the specificity of the antibody. σ^A^ levels were monitored as a loading control. The position of σ^G^ and σ^A^ is indicated by arrowheads.
(EPS)

Figure S3 Time-lapse microscopy of P*_sspE_*-*cfp*. Sporulating cells were incubated on agarose pads at 30°C. Images sequences were initiated 2 hours after the onset of sporulation in DSM and the first image was set to t = 0 min. Minutes after t = 0 is indicated in the lower right corner of each CFP image. The upper panels show phase contrast; the lower panels show whole cell CFP. The arrow indicates a cell with whole cell CFP accumulation.
(EPS)

Figure S4 Panel A: The *B. subtilis* CsfB protein shows structural similarity to several proteins containing zinc finger domains. Several of these proteins belong to the family of nuclear hormone receptor transcriptional regulators, with one of the most significant hits to the human vitamin D3 receptor protein (pdb code: 1kb2). The critical Cys are shown in blue (numbering is from the beginning of the sequences shown); other identical (yellow) or conserved residues (green) are also highlighted. Panel B: an *E. coli* strain expressing a C-terminal fusion of the *Strep* II tag to CsfB under the control of the T7*lac* promoter was grown in mininal medium in the presence of iron (lanes 1–3) or zinc (lanes 4–6) and induced with 1 mM IPTG for 2 hours. Lanes are as follows: 1 and 4, total extract; 2 and 5, insoluble fraction; 3 and 6, soluble fraction. Panel C depicts the induction of CsfB-*Strep* II tag by an auto-induction regime, and its purification. A strain with an “empty” vector is used as a control for the auto-induction (lanes 1, 3, and 5). Lanes are as follows: 1 and 2, total extract; 3 and 4, insoluble fraction; 5 and 6, soluble fraction; 7, protein (CsfB-*Strep* II tag) purified after a after a streptavidin affinity column. Panel D depicts the time-course of oxidant-induced (1 mM H_2_O_2_) zinc release by purified CsfB, as monitored after reaction with 4-(2-pyridylazo) resorcinol (PAR), by measuring the OD at 500 nm. No zinc is released when the protein is kept reduced in the presence of DTT.
(EPS)

Figure S5 Panel A: growth curves for strain AH6689 in LB containing the indicated xylose concentrations. Panel B: expression of P*_sspE_-cfp* and *csfB-yfp* was monitored in the same cells, by fluorescence microscopy, at the onset of stationary phase in LB. The strain used (AH6689) additionally carries a deletion of the *sigG* gene, and a second copy of the wild type *sigG* gene under the control of the xylose inducible P*_xylA_* promoter inserted at the *amyE* locus. Cells were were grown in the presence of different concentrations of xylose, as indicated (NB: images obtained in the presence of 0.0001% xylose are not represented for simplicity). Scale bar, 2 µm. Panel C: quantitative analysis of CFP and YFP expression for the AH6689 strain (as in panel B), at the xylose concentrations indicated in panel A. The top graph shows the correlation between the YFP (*csfB-yfp*) and CFP (P*_sspE_-cfp*) signals for the various concentrations of xylose. The middle and bottom graph show a cumulative frequency distribution of the YFP and CFP signals across the population. In all three panels the fluorescence intensity is shown in arbitrary units; 100 cells were scored. The legend applies to all three graphs.
(EPS)

Figure S6 Panel A shows the crystal structure of the *Thermus aquaticus* σ^70^-containing RNA polymerase holoenzyme (RNAP), drawn with PyMol (www.pymol.org) from the coordinates reported by Murakami *et al.* (2002) [45]. The two α subunits are shown one in blue, the other in purple, β is colored grey, β′ is shown in green, and σ^70^ in yellow. The region encircled, part of the σ^70^/β′ interface, is magnified in Panel B. Here, the contact between E189 in the σ^70^ subunit and R159 in β′ can be clearly seen. Other amino acids located close to the E189 residue and contributing to the interaction are represented, as are the distances (in Å) between them. Residue E189 is equivalent to N45 in σ^G^ and E39 in σ^F^.
(EPS)

Table S1 *Bacillus subtilis* strains used in this work.
(PDF)

Table S2 Oligonucleotide primers used in this work.
(PDF)

Text S1 Supporting Materials and Methods, Results and Discussion.
(DOC)
