# Supplementary material for: A Negative Feedback Loop That Limits the Ectopic Activation of a Cell Type–Specific Sporulation Sigma Factor of Bacillus subtilis
Source: PLoS Genet. 2011 Sep 15;7(9):e1002220. doi: 10.1371/journal.pgen.1002220 (PMC3174212; doi:10.1371/journal.pgen.1002220)
Supplement: Table S1 — Bacillus subtilis strains used in this work. (PDF) [file pgen.1002220.s007.pdf]

Table S1 - *Bacillus subtilis* strains used in this work.

Strain	Relevant Genotype	Origin
MB24	trpC2 metC3	Laboratory stock
AH1042	trpC2 metC3 ΔsspE::P_{sspE}-lacZ	“
AH2357	trpC2 metC3 ΔsigF::erm	“
AH2452	trpC2 ΔsigG ΔsspE::P_{sspE}-lacZ	[1]
AH2492	trpC2 ΔsigG ΔsspE::P_{sspE}-lacZ ΔamyE::P_{xylA}-sigG ΔthrC::P_{spac}-spoIIAB	«
AH2493	trpC2 ΔsigG ΔsspE::P_{sspE}-lacZ ΔamyE::P_{xylA}-sigGE156K ΔthrC::P_{spac}-spoIIAB	«
AH3447	trpC2 metC3 spoIIQΩspoIIQ-lacZ	“
AH3795	trpC2 ΔsigG	[1]
AH6513	trpC2 ΔcsfB::km ΔsigG ΔsspE::P_{sspE}-lacZ ΔyycR::P_{sspE}-cfp	This work
AH6539	trpC2 sigGF91AY94A ΔyycR::P_{sspE}-cfp ΔsspE::P_{sspE}-lacZ	«
AH6556	trpC2 ΔsigG ΔsspE::P_{sspE}-lacZ ΔamyE::P_{xylA}-sigGN45E ΔthrC::P_{spac}-spoIIAB	«
AH6566	trpC2 ΔsigG ΔsspE::PsspE-lacZ ΔyycR::P_{sspE}-cfp	[11]
AH6567	trpC2 ΔsspE::P_{sspE}-lacZ ΔyycR::P_{sspE}-cfp	This work
AH6568	trpC2 metC3 ΔcsfB::km ΔsspE::P_{sspE}-lacZ ΔyycR::P_{sspE}-cfp	«
AH6574	trpC2 sigGN45E ΔsspE::P_{sspE}-lacZ ΔyycR::P_{sspE}-cfp	«
AH6575	trpC2 ΔcsfB::km sigGN45E ΔsspE::P_{sspE}-lacZ ΔyycR::P_{sspE}-cfp	«
AH6581	trpC2 metC3 ΔspoIIIJ::sp ΔsspE::P_{sspE}-lacZ ΔyycR::P_{sspE}-cfp	«
AH6584	trpC2 metC3 ΔcsfB::km ΔspoIIIJ::sp ΔsspE::P_{sspE}-lacZ ΔyycR::P_{sspE}-cfp	«
AH6587	trpC2 ΔspoIIIJ::sp sigGN45E ΔsspE::P_{sspE}-lacZ ΔyycR::P_{sspE}-cfp	«
AH6608	trpC2 metC3 ΔcsfB::km ΔsigG ΔamyE::csfB-gfp	«
AH6609	trpC2 sigGN45A ΔsspE::P_{sspE}-lacZ ΔyycR::P_{sspE}-cfp	«
AH6610	trpC2 sigGN45E spoIIQΩspoIIQ-lacZ	«
AH6614	trpC2 ΔyycR::P_{sspE}-cfp ΔcsfB::csfB-yfp	«
AH6617	trpC2 sigGN45E ΔyycR::P_{sspE}-cfp ΔcsfB::csfB-yfp	«
AH6625	trpC2 sigGN45E ΔyycR::P_{sspE}-cfp ΔsspE::P_{sspE}-lacZ sigF::erm	«
AH6626	trpC2 sigGN45E ΔyycR::P_{sspE}-cfp ΔsspE::P_{sspE}-lacZ spo0A::neo	«
AH6633	trpC2 sigGK5D ΔsspE::P_{sspE}-lacZ ΔyycR::P_{sspE}-cfp	«
AH6634	trpC2 sigGC9K ΔsspE::P_{sspE}-lacZ ΔyycR::P_{sspE}-cfp	«
AH6635	trpC2 sigGΔ10-11 ΔsspE::P_{sspE}-lacZ ΔyycR::P_{sspE}-cfp	«
AH6636	trpC2 sigGD12K ΔsspE::P_{sspE}-lacZ ΔyycR::P_{sspE}-cfp	«
AH6637	trpC2 sigGT13N ΔsspE::P_{sspE}-lacZ ΔyycR::P_{sspE}-cfp	«
AH6638	trpC2 sigGL16N ΔsspE::P_{sspE}-lacZ ΔyycR::P_{sspE}-cfp	«
AH6639	trpC2 sigGV18Q ΔsspE::P_{sspE}-lacZ ΔyycR::P_{sspE}-cfp	«
AH6640	trpC2 sigGN21D ΔsspE::P_{sspE}-lacZ ΔyycR::P_{sspE}-cfp	«
AH6641	trpC2 sigGM24V ΔsspE::P_{sspE}-lacZ ΔyycR::P_{sspE}-cfp	«
AH6642	trpC2 sigGR25K ΔsspE::P_{sspE}-lacZ ΔyycR::P_{sspE}-cfp	«
AH6643	trpC2 sigGF28I ΔsspE::P_{sspE}-lacZ ΔyycR::P_{sspE}-cfp	«
AH6644	trpC2 sigGD33N ΔsspE::P_{sspE}-lacZ ΔyycR::P_{sspE}-cfp	«
AH6645	trpC2 sigGD37Q ΔsspE::P_{sspE}-lacZ ΔyycR::P_{sspE}-cfp	«
AH6646	trpC2 sigGS38Q ΔsspE::P_{sspE}-lacZ ΔyycR::P_{sspE}-cfp	«
AH6647	trpC2 sigGK42L ΔsspE::P_{sspE}-lacZ ΔyycR::P_{sspE}-cfp	«
AH6648	trpC2 sigGV44I ΔsspE::P_{sspE}-lacZ ΔyycR::P_{sspE}-cfp	«
AH6649	trpC2 sigGG46K ΔsspE::P_{sspE}-lacZ ΔyycR::P_{sspE}-cfp	«
AH6650	trpC2 sigGL48M ΔsspE::P_{sspE}-lacZ ΔyycR::P_{sspE}-cfp	«
AH6651	trpC2 sigGL52W ΔsspE::P_{sspE}-lacZ ΔyycR::P_{sspE}-cfp	«
AH6652	trpC2 sigGN59L ΔsspE::P_{sspE}-lacZ ΔyycR::P_{sspE}-cfp	«
AH6653	trpC2 sigGE63Y ΔsspE::P_{sspE}-lacZ ΔyycR::P_{sspE}-cfp	«
AH6654	trpC2 sigGY64E ΔsspE::P_{sspE}-lacZ ΔyycR::P_{sspE}-cfp	«
AH6655	trpC2 sigGV65P ΔsspE::P_{sspE}-lacZ ΔyycR::P_{sspE}-cfp	«
AH6656	trpC2 sigGV71I ΔsspE::P_{sspE}-lacZ ΔyycR::P_{sspE}-cfp	«
AH6657	trpC2 sigGM77L ΔsspE::P_{sspE}-lacZ ΔyycR::P_{sspE}-cfp	«
AH6658	trpC2 sigGN82K ΔsspE::P_{sspE}-lacZ ΔyycR::P_{sspE}-cfp	«
AH6678	trpC2 sigGF91AY94A ΔyycR::P_{sspE}-cfp ΔcsfB::csfB-yfp	«
AH6679	trpC2 sigGN45EF91AY94A ΔyycR::P_{sspE}-cfp ΔcsfB::csfB-yfp	«
AH6680	trpC2 sigGN45E ΔyycR::P_{sspE}-cfp ΔcsfB::csfB-yfp sigF::erm	«
AH6686	trpC2 metC3 ΔsigF::erm ΔthrC::P_{spac}-csfB ΔamyE::spoIIAABCwt	«
AH6687	trpC2 metC3 ΔsigF::erm ΔthrC::P_{spac}-csfB ΔamyE::spoIIAABC E39N	«
AH6688	trpC2 ΔsigG ΔyycR::P_{sspE}-cfp ΔcsfB::csfB-yfp ΔamyE::P_{xylA}-sigGE156K	«

AH6689	*trpC2* Δ*sigG* Δ*yycR*::P_{sspE}-*cfp* Δ*csfB*::*csfB-yfp* Δ*amyE*::P_{xylA}-*sigGwt*	«
AH6690	*trpC2* Δ*sigG* Δ*yycR*::P_{sspE}-*cfp* Δ*csfB*::*csfB-yfp* Δ*amyE*::P_{xylA}-*sigGN45E*	«
AH6691	*trpC2 metC3* Δ*sigF*::*erm* Δ*thrC*::P_{spac}-*csfB* Δ*amyE*::*spoIIAABCwt yuiC*::P_{yuiC}-*gfp*	«
AH6692	*trpC2 metC3* Δ*sigF*::*erm* Δ*thrC*::P_{spac}-*csfB* Δ*amyE*::*spoIIAABC E39N yuiC*::P_{yuiC}-*gfp*	«
AH6698	*trpC2* Δ*sigG* Δ*yycR*::P_{sspE}-*cfp* Δ*csfB*::*csfB-yfp* Δ*amyE*::P_{xylA}*sigGwt* Δ*lonA*::*cat*	«
AH6723	*trpC2* Δ*sigG csfB*::*csfB-lacZ* Δ*amyE*::P_{xylA}-*sigGwt*	"
AH6724	*trpC2* Δ*sigG csfB*::P_{csfB}-*lacZ* Δ*amyE*::P_{xylA}-*sigGwt sigF*::*erm*	"
AH6789	*trpC2* Δ*sigG* Δ*yycR*::P_{sspE}-*cfp* Δ*csfB*::*csfB-yfp* Δ*amyE*::P_{xylA}-*sigGN45E/E156K*	«
BTD2633	Δ*yycR*::P_{sspE}-*cfp*	D. Rudner
JOB20	*trpC2 metC3* Δ*spoIIIJ*::*sp*	[12]
MO3632	Δ*csfB*::*km*	P. Stragier
SL14127	Δ*thrC*::P_{spac}-*csfB* Δ*amyE*::P_{gerE}-*lacZ*	P. Piggot
