# Supplementary material for: A Negative Feedback Loop That Limits the Ectopic Activation of a Cell Type–Specific Sporulation Sigma Factor of Bacillus subtilis
Source: PLoS Genet. 2011 Sep 15;7(9):e1002220. doi: 10.1371/journal.pgen.1002220 (PMC3174212; doi:10.1371/journal.pgen.1002220)
Supplement: Table S2 — Oligonucleotide primers used in this work. (PDF) [file pgen.1002220.s008.pdf]

Table S2 – Oligonucleotide primers used in this work.

Primer	**Sequence (5′ → 3′)**
csfB12D	CGCCGCCAAGCAAGCTTAAACCCAGC
csfB191D	GGTGGAGACCATGGACGAAACAG
csfB681D	CCATGAATTCATAGACCTGAAAAGGTC
csfB480R	ATTATTATTGTCGACTACGTTCAATCC
csfB1146R	CTCAGGATCCTTAGCATTTCTGCATTTTGC
csfBgfpR	GTTCTTCTGGTTTACTGTTGTTGTTGTTTGAATATAATGGCGGTGTATG
csfBstrepR	CGGGATCCTTATTTTTCGAACTGCGGGTGGCTCCAAGCGCTTGAATATAATGGCGGTGTAT[illegible]
gfp30D	AGTAAAGGAGAAGAACTTTTCACTGGAG
gfpR	GGCGGATCCTTATTTGTATAGTTCATCCATGC
yfpR	GCATGCCTGCAGGTCTGGAC
sigF493D	GATTTGCCATGGATGTGGAGG
sigF219D	GAAGGATCCGAAGAAAGCCGGG
sigF1318R	GACCATGAATTCCCACGCTTTTGC
sigF2032R	GATTAAGCTTCTCATTCATCCGCTCG
sigFE39ND	GAGACCTCCTCATAAACAAAAACATGCGTC
sigFE39NR	GACGCATGTTTTTGTTTATGAGGAGGTCTC
sigG2016D	GGGAAAACCATGGCGAGAAATAAAGTCG
sigG2862R	CCATCCAGAATTCAATAGAAAAGCC
sigG2964R	GCAAAATCCTCGAGGTCCTCCTCTTTATTGCC
sigGK5DD	GTGTCGAGAAATGATGTCGAAATCTGCGGG
sigGK5DR	CCCGCAGATTTCGACATCATTTCTCGACAC
sigGC9KD	GAAATAAAGTCGAAATCAAAGGGGTGGATACCTCC
sigGC9KR	GGAGGTATCCACCCCTTTGATTTCGACTTTATTTC
sigGΔ10-11D	GATACCTCCAAATTACCAGTACTC
sigGΔ10-11R	GCAGATTTCGACTTTATTTCTCG
sigGD12KD	CGAAATCTGCGGGGTGAAAACCTCCAAATTACC
sigGD12KR	GGTAATTTGGAGGTTTTCACCCCGCAGATTTCG
sigGT13ND	CTGCGGGGTGGATAACTCCAAATTACCAG
sigGT13NR	CTGGTAATTTGGAGTTATCCACCCCGCAG
sigGL16ND	GGTGGATACCTCCAAAAATCCAGTACTCAAGAATG
sigGL16NR	CATTCTTGAGTACTGGATTTTTGGAGGTATCCACC
sigGV18QD	CCTCCAAATTACCACAACTCAAGAATGAAGAG
sigGV18QR	CTCTTCATTCTTGAGTTGTGGTAATTTGGAGG
sigGN21DD	CCAGTACTCAAGGATGAAGAGATGAGAAAGC
sigGN21DR	GCTTTCTCATCTCTTCATCCTTGAGTACTGG
sigGM24VD	CTCAAGAATGAAGAGGTGAGAAAGCTGTTTAGGC
sigGM24VR	GCCTAAACAGCTTTCTCACCTCTTCATTCTTGAG
sigGR25KD	GAATGAAGAGATGAAAAAGCTGTTTAGGC
sigGR25KR	GCCTAAACAGCTTTTTCATCTCTTCATTC
sigGF28ID	GAGATGAGAAAGCTGATTAGGCAGCTGCAGG
sigGF28IR	CCTGCAGCTGCCTAATCAGCTTTCTCATCTC
sigGD33ND	GTTTAGGCAGCTGCAGAATGAAGGCGATGATTCAGC
sigGD33NR	GCTGAATCATCGCCTTCATTCTGCAGCTGCCTAAAC
sigGD37QD	GCAGGATGAAGGCGATCAATCAGCAAGAGAAAAG
sigGD37QR	CTTTTCTCTTGCTGATTGATCGCCTTCATCCTGC
sigGS38QD	GGATGAAGGCGATGATCAAGCAAGAGAAAAGC
sigGS38QR	GCTTTCTCTTGCTTGATCATCGCCTTCATCC
sigGK42LD	GATTCAGCAAGAGAATATCTTGTAAACGGGAAC
sigGK42LR	GTTCCCGTTTACAAGATATTCTCTTGCTGAATC
sigGV44ID	GCAAGAGAAAAGCTTATAAACGGGAACTTGCG
sigGV44IR	CGCAAGTTCCCGTTTATAAGCTTTTCTCTTGC
sigGN45ED	GAGAAAAGCTTGTAGAGGGGAACTTGCGTCTTC
sigGN45ER	GAAGACGCAAGTTCCCCTCTACAAGCTTTTCTC
sigGN45AD	GAGAAAAGCTTGTAGCCGGGAACTTGCGTCTTG
sigGN45AR	CAAGACGCAAGCTCCCGGCTACAAGCTTTTCTC
sigGG46KD	GAAAAGCTTGTAAACAAGAACTTGCGTCTTG
sigGG46KR	CAAGACGCAAGTTCTTGTTTACAAGCTTTTC
sigGL48MD	GTAAACGGGAACATGCGTCTTGTCTTAAGTG
sigGL48MR	CACTTAAGACAAGACGCATGTTCCCGTTTAC

sigGL52WD	GGAACTTGCGTCTTGTCTGGAGTGTCATTCAACG
sigGL52WR	CGTTGAATGACACTCCAGACAAGACGCAAGTTCC
sigGN59LD	GTCATTCAACGATTTCTTAACAGAGGGGAG
sigGN59LR	CTCCCCTCTGTTAAGAAATCGTTGAATGAC
sigGE63YD	CGATTTAATAACAGAGGGTATTATGTTGATGAC
sigGE63YR	GTCATCAACATAATACCCTCTGTAATTAAATCG
sigGY64ED	CAGAGGGGAGGAAGTTGATGAC
sigGY64ER	GTCATCAACTACCTCCCCTCTG
sigGV65PD	CAGAGGGGAGTATCCTGATGACTTATTTC
sigGV65PR	GAAATAAGTCATCAGGATACTCCCCTCTG
sigGV71ID	GACTTATTTCAAGCCGGCTGCATCGGAC
sigGV71IR	GTCCGATGCAGCCGGCTTGAAATAAGT
sigGM77LD	CTGCATCGGACTATTGAAATCCATTG
sigGM77LR	CAATGGATTTCAATAGTCCGATGCAG
sigGN82KD	GAATCCATTGATAAATTTGACCTAAGCCAC
sigGN82KR	GTGGCTTAGGTCAAATTTATCAATGGATTTC
sigGF91AY94AD	GCCACAATGTTAAGGCTTCAACAGCCGCTGTACCTATGATTATCGG
sigGF91AY94AR	CCGATAATCATAGGTACAGCGGCTGTTGAAGCCTTAACATTGTGGC
spoIIAB189D	GGGGGTGGATCCATGAAAAATGAAATGC
spoIIAB698R	CCTTCAGCTCGAGCGTTTTTGCCG
csfB578R	CGTATAAAGATGGATCCTCTATTCTTCTC
