# Supplementary material for: A Negative Feedback Loop That Limits the Ectopic Activation of a Cell Type–Specific Sporulation Sigma Factor of Bacillus subtilis
Source: PLoS Genet. 2011 Sep 15;7(9):e1002220. doi: 10.1371/journal.pgen.1002220 (PMC3174212; doi:10.1371/journal.pgen.1002220)
Supplement: Text S1 — Supporting Materials and Methods, Results and Discussion. (DOC) [file pgen.1002220.s009.doc]

**Text S1**

**Strains and general methods.** The *B. subtilis* strains included in the supporting information are congenic derivatives of the Spo^+^ strain MB24 (*trpC2 metC3*), and are listed with their complete genotype in Table S1. The plasmids used in strain construction are described in the sections below. LB medium was used for routine growth or maintenance of *E. coli* and *B. subtilis*, and sporulation was induced by growth and exhaustion in Difco sporulation medium (DSM) [1]. The Quick Change site-directed mutagenesis system (Stratagene) was used for the generation of all site-specific mutations, which were always confirmed by sequencing.

**Fusion of the *xylA* promoter to different *sigG* alleles**. Fusion of the *xylA* promoter to the N45E allele of *sigG* was made using pMS237, containing the *xylA* promoter region and the 5’ end of the *sigG* gene [1] and primers sigGN45ED and sigGN45ER (all primers used are listed in Table S2) were used to substitute the asparagine codon at position 45 of *sigG* for a glutamate codon, which resulted in plasmid pJS16. pJS16 was used to re-construct a copy of the *sigG* gene carrying the N45E or both the N45E/E156K mutations at *amyE* under the control of the *xylA* promoter, as previously described for the wild type and the E156K allele [1].

**A *csfB-gfp* fusion at *amyE* and a *csfB-yfp* fusion at the *csfB* locus.** A *csfB-gfp* fusion to be integrated at the non-essential *amyE* locus was constructed in two steps. Initially, the *csfB* and *gfp* sequences were amplified separately from chromosomal DNA of a wild type *B. subtilis* strain and from pEA18 (a gift from Alan Grossman), using primer pair csfB12D/csfB-gfpR or gfp30D/gfpR, respectively. Second, the 380 bp *csfB* fragment and the 719 bp *gfp* fragments were mixed together and amplified using primers csfB12D and *gfp*-R. Finally, the resulting 1099 bp *csfB-gfp* fragment was cloned between the *Hin*dIII and *Bam*HI sites of pDG364 [2], yielding pMS261.

A *csfB-yfp* fusion to be transferred to the *csfB* locus was constructed in two steps. First, the *csfB* and *yfp* sequences were amplified separately from chromosomal DNA of a wild type *B. subtilis* strain and from pKL183 (a gift from Alan Grossman), with primer pairs csfB12D/csfB-gfpR and gfp30D/yfpR, respectively. The 380 bp *csfB* fragment and the 719 bp *yfp* fragment were mixed together and amplified using primers csfB12D and *yfp*-R. The resulting 1099 bp *csfB-yfp* fragment was introduced between the *Hin*dIII and *Sph*I sites of pAH250 [3], to yield pMS382. Second, a 465pb DNA fragment, downstream of the *csfB* coding region, was PCR amplified with primers *csfB*681D and *csfB*1146R, digested with *Eco*RI and *Bam*HI and inserted between the same sites of pMS382, yielding pMS383.

**Construction of a P*_csfB_-lacZ* fusion.** To construct a P*_csfB_-lacZ* fusion to be integrated at the *csfB* locus the upstream region was amplified from chromosomal DNA of a wild type *B. subtilis* strain using the primer csfB12D and csfB578R. The resulting 266 bp *csfB* fragment was cloned between the *Sma*I and *Bam*HI sites of pJM783 [4], to yield pMS412.

**Construction of the *sigG F91AY94A* mutant.** We used primers *sigGF91AY94AD* and *sigGF91AY94AR* (Table 2), to simultaneously convert codons 91 and 94 of *sigG* (specifying phenylalanine and tyrosine, respectively) into alanine codons. The mutations were introduced into pMS45, which carries the wild type *sigG* gene [1], producing pMS291. The mutations were then transferred to the *sigG* locus by congression as follows: pMS291, together with chromosomal DNA from AH6567 (D*yycR:*:P*_sspE_*-*cfp* D*sspE*::P*_sspE_*-*lacZ*), were used to co-transform strain AH1042 (D*sspE*::P*_sspE_*-*lacZ*) with selection to Cm^R^ . Spo^-^ congressants appeared at a frequency of about 3%. Spo^-^ congressants from each of the transformations, shown by PCR and sequence analysis to carry the desired mutations in *sigG*, were chosen for further study and named AH6539 (F91A and Y94A) (Table S1).

**Time-lapse microscopy.** Time-lapse experiments were conducted as described before [5]. Briefly, cells were grown in DSM until one hour after the onset of sporulation. A 2-ml aliquot was taken, the cells sedimented by centrifugation, and the supernatant used to make a 1.2% agarose pad. The culture was spread on this pad, covered with a glass coverslip, and equilibrated to 37ºC for 10 min prio to viewing. Cells were maintained at 37ºC using a Pecon objective heater (www.pecon.biz) and imaged at the indicated times, in minutes (Figure S3).

**PAR assay.** 10 µg of purified CsfB protein were incubated in the absence or in the presence of H_2_O_2_ (1 and 10 mM, in parallel reactions) and samples taken at time intervals for measurement of the released Zn^2+^ (Figure S4). Released Zn^2+^ was quantified by reacting samples taken from the reaction vessel with 0.1 mM of the metal chelator 4-(2-pyridylazo) resorcinol (PAR) and recording the absorbance at 500 nm. The absorbance was converted to the amount of released zinc using a standard curve prepared with known amounts of ZnCl_2_, as described before [6].

**Molecular imaging.** Figures of the *Thermus aquaticus* RNA polymerase, s^A^-containing holoenzyme were produced using PyMol ([www.pymol.org](http://www.pymol.org/)) using the coordinates (PDB accession number 1L9Z) reported by Murakami *et al*. (2002). Distances between residues involved in the contact depicted (Figure S7) between the relevant residues in the b´and s subunits are shown in Å.

Results and Discussion

**An inactive, “promoter-melting” mutant of s^G^**.

Several aromatic residues in region 2.1 of sigma factors of the σ^70^ family have been implicated in melting of the promoter DNA during transcription initiation (reviewed by [7]). These residues tend not to be conserved in alternative sigma factors where the capacity to melt promoters is traded for increased stringency in promoter recognition. However, residues homologous to F431/F86 and Y434/Y89 of the *E. coli* σ^70^ and the *B. subtilis* σ^A^ proteins, respectively, which have been directly implicated in promoter melting, are conserved in σ^G^ (Figure S1A). These residues in σ^G^, F91 and Y94, were both changed to an alanine. The double mutant did not support sporulation (not shown) and was unable to direct transcription of the σ^G^-responsive fusion of the *sspE* promoter to the *lacZ* gene. Panel B of figure 1 shows the induction of P*_sspE_-lacZ* expression during sporulation of a wild type strain in Difco sporulation medium (DSM). σ^G^ becomes active when engulfment of the forespore by the mother cell comes to an end, and this coincides, under our experimental conditions, with hour 3 of sporulation. The mutant form of σ^G^ (s^GF91A/Y94A^) is unable to activate transcription of P*_sspE_-lacZ* (Figure S1B). Nevertheless, the protein accumulates in whole cell extracts prepared in parallel (Figure S1C). The levels of σ^G^ in the *sigGF91AY94A* mutant are therefore a measure of the production of σ^G^ under the control of σ^F^, in the absence of the σ^G^ auto-regulatory loop normally activated following engulfment completion.

**The N45E substitution in σ^G^ does not seem to titrate a negative regulator of σ^F^**.

Because the two forespore-specific sigma factors σ^F^ and σ^G^ have partially overlapping promoter specificities, one possible explanation for the increased expression of the *sspE-lacZ* reporter fusion would be that the N45E mutation somehow caused the mutant form of σ^G^ to titrate a negative regulator of σ^F^, partially releasing the latter from inhibition under non-sporulation conditions, or even during stationary phase in DSM medium, prior to asymmetric division. If so, then the N45E substitution in σ^G^ should cause an increase in the activity of σ^F^. We tested this possibility by monitoring the expression of a fusion of the promoter for the *spoIIQ* gene, known from previous studies to be under the control of σ^F^. The N45E substitution in σ^G^ did not alter the activity of σ^F^ (at least when measured with the *spoIIQ-lacZ* reporter), as expression of *spoIIQ-lacZ* was similar in the wt or N45E mutant (Figure S2A). Together with additional observations (see main text), this result therefore strengthens the view that the increased expression of *sspE-lacZ* in the N45E mutation was indeed caused by augmented activity of σ^G^.

**The N45E substitution does not make σ^G^ resistant to SpoIIAB.**

As a test to determine whether σ^GN45E^ remained sensitive to SpoIIAB in vivo, we constructed strains engineered to co-express *spoIIAB* and either *sigG*, *sigGN45E*, or *sigGE156K* (coding for a form of σ^G^ that is not efficiently bound by SpoIIAB; [1]), during vegetative growth in LB medium. In these strains the *sigG*, *sigGN45E* or *sigGE156K* alleles were inserted at the non-essential *amyE* locus under the control of the xylose-inducible P*_xylA_* promoter, whereas an IPTG-inducible P*_spac_*-*spoIIAB* fusion was inserted at the non-vital *thrC* locus. In addition, all the strains carried a P*_sspE_-lacZ* reporter fusion inserted at the *sspE* locus (see Table 1). We then monitored the P*_sspE_*-*lacZ*-driven production of β-galactosidase in the various cultures during the exponential phase of growth and at the onset of stationary phase in LB medium (1.5, 2 and 2.5 h after inoculation). Note that in these experiments, basal expression of the *sigG* alleles from the non-induced P*_xylA_* promoter was sufficient to detect P*_sspE_*-*lacZ* expression. As expected, the activity of σ^GE156K^ was insensitive to the induction of SpoIIAB production with IPTG (Figure S2B) [1]. In contrast, the activity of the wild type and N45E forms of σ^G^ was immediately reduced to ~50% of the level observed in the absence of SpoIIAB, and remained at low levels ([1]; Figure S2B). Thus, both the GST pull-down experiments illustrated in Figure 4C, and the activity assay described above suggest that the N45E substitution does not significantly interfere with binding of SpoIIAB to σ^G^.

**Stationary phase cells in sporulation medium that show active σ^G^ undergo lysis**.

The stationary phase cells in sporulation medium (DSM) that showed increased activity of σ^G^ did not show any morphological signs of sporulation. Specifically, asymmetric septa were never seen. The sub-population of σ^G^-active cells could represent a sort of “persister cells” that did not enter sporulation but remained viable. We used time-lapse microscopy to follow the fate of N45E cells, expressing a P*_sspE_-cfp* fusion as a reporter for σ^G^ activity. This analysis (show in figure S3 are examples of two cells of several examined with a similar fate), indicated that these cells eventually undergo lysis, as seen by their appearance under phase contrast optics. Lysis is concomitant with the loss of the CFP signal.

**Purification and properties of the CsfB protein**.

CsfB is a novel type of Zn^2+^-containing anti-sigma factor [8,9]. The protein shows similarity to several proteins with zinc finger domains, and the highest structural similarity to the human vitamin D3 receptor protein (pdb code 1kb2), which belongs to the family of nuclear hormone receptor transcriptional regulators, as shown (Figure S4, panel A). These proteins are marked by the presence of two Cys-X[2]-Cys motifs which form a C4-type zinc finger, and are grouped in Pfam family PF00105 (<http://pfam.sanger.ac.uk/family?acc=PF00105>). The C4-type zinc finger domain is often involved in protein-protein interactions. The alignment highlights the conservancy of the 4 C residues between the two proteins. Other functionally important residues or regions of CsfB as defined by Rhayat *et al*. (2009) are not indicated. Consistent with its ability to bind Zn^2+^, previously shown by Rhayat *et al*. (2009), overproduction of a CsfB-*Strep* II tag fusion protein from the T7*lac* promoter in *E. coli* cells grown in a minimal medium, only resulted in soluble protein when Zn^2+^ (and not iron, for example) was added (Figure S4B). The protein could then be purified in high yield and purity from cultures in LB medium (which is rich in Zn^2+^), upon IPTG induction (Figure S4C). The bound Zn^2+^( Zn^2+^to protein ratio of 1:1 as determined by atomic absorption) could be released from the protein upon oxidation with H_2_O_2_ (Figure S4D) suggesting that binding involved the C residues in CsfB.

**Induction of σ^G^ under non-sporulation conditions**.

Expression of P*_sspE_-cfp* and *csfB-yfp* was quantified in individual cells, by fluorescence microscopy as a function of the xylose concentration used to induce production of wild type σ^G^ (Figure S5B). The results show a good correlation between the YFP and CFP signals at all the xylose concentrations used (Figure S6C, top). Cells that produce YFP also make CFP and the higher the level of σ^G^, the higher the production of both reporters. Moreover, the higher the production of wild type σ^G^, the higher the representation of cells with high levels of YFP and CFP fluorescence, as shown in the cumulative frequency distributions (Figure S5C, middle and bottom graphs). Cell lysis occurs for high levels (0.01 and 0.1%) of the xylose inducer (Figure S5A), and is related to high levels of σ^G^ activity. Possibly, there is a threshold level above which the activity of σ^G^ directly or indirectly causes lysis.

**Residue N45 in region 2.1 of σ^G^ is likely to participate in a direct contact with the β´subunit of RNA polymerase.**

The N45 residue in σ^G^ is homologous to E189 in the σ^70^ homologue of *Thermus aquaticus* [10]. The structure of the *T. aquaticus* holoenzyme (Figure S6A) reveals that E189 participates in a contact with the β´subunit of RNA polymerase, together with H186 and E87 (Figure S7B). Even though the σ^G^ protein is smaller and could not be correctly modeled as several elements of secondary structure around the N45 position are missing relative to the σ^70^ protein of *T. aquaticus* (namely elements that contain the H186 and E87 residues of *T. aquaticus* σ^70^), it seems plausible that the N45 residue also participates in a contact with the β´subunit. This assumption is in line with the localization of N45 within conserved region 2.1, implicated in core binding, and also with one general mechanism by which anti-sigma factors negatively regulate their cognate σ, that is, by occluding a σ/core interaction site. If so, then the N45E substitution while interfering with binding of the CsfB anti-sigma factor to σ^G^, may additionally promote the interaction with the catalytic core of RNA polymerase.

References

1. Serrano M, Neves A, Soares CM, Moran CP, Jr., Henriques AO (2004) Role of the anti-sigma factor SpoIIAB in regulation of sigmaG during Bacillus subtilis sporulation. J Bacteriol 186: 4000-4013.

2. Cutting SaHPB (1990) Genetic Analysis; Harwood CRaCSM, editor: John Wiley and sons, Lta.

3. Henriques AO, Beall BW, Moran CP, Jr. (1997) CotM of Bacillus subtilis, a member of the alpha-crystallin family of stress proteins, is induced during development and participates in spore outer coat formation. J Bacteriol 179: 1887-1897.

4. Perego M, Hoch JA (1987) Isolation and sequence of the spo0E gene: its role in initiation of sporulation in Bacillus subtilis. Mol Microbiol 1: 125-132.

5. Gutierrez J, Smith R, Pogliano K SpoIID-mediated peptidoglycan degradation is required throughout engulfment during Bacillus subtilis sporulation. J Bacteriol 192: 3174-3186.

6. Bae JB, Park JH, Hahn MY, Kim MS, Roe JH (2004) Redox-dependent changes in RsrA, an anti-sigma factor in Streptomyces coelicolor: zinc release and disulfide bond formation. J Mol Biol 335: 425-435.

7. Feklistov A, Darst SA (2009) Promoter recognition by bacterial alternative sigma factors: the price of high selectivity? Genes Dev 23: 2371-2375.

8. Karmazyn-Campelli C, Rhayat L, Carballido-Lopez R, Duperrier S, Frandsen N, et al. (2008) How the early sporulation sigma factor sigmaF delays the switch to late development in Bacillus subtilis. Mol Microbiol 67: 1169-1180.

9. Rhayat L, Duperrier S, Carballido-Lopez R, Pellegrini O, Stragier P (2009) Genetic dissection of an inhibitor of the sporulation sigma factor sigma(G). J Mol Biol 390: 835-844.

10. Murakami KS, Masuda S, Darst SA (2002) Structural basis of transcription initiation: RNA polymerase holoenzyme at 4 A resolution. Science 296: 1280-1284.

11. Serrano M, Vieira F, Moran CP, Jr., Henriques AO (2008) Processing of a membrane protein required for cell-to-cell signaling during endospore formation in Bacillus subtilis. J Bacteriol 190: 7786-7796.

12. Serrano M, Corte L, Opdyke J, Moran CP, Jr., Henriques AO (2003) Expression of spoIIIJ in the prespore is sufficient for activation of sigma G and for sporulation in Bacillus subtilis. J Bacteriol 185: 3905-3917.

Supplemental figures legends

**Figure S1.** Panel A shows the sequence alignment of the subregion 2.3 of σ^G^ from *B. subtilis* with the same region of σ^A^ from *B. subtilis* and σ^70^ from *E. coli*. The aminoacids (F91 and Y94) of σ^G^ changed to alanine are highlighted. Panel B shows the expression of a σ^G^-dependent P*_sspE_-lacZ* fusion during stationary phase in DSM in the following strains: wild type background (AH6567, Δ*yycR:*:P*_sspE_*-*cfp* Δ*sspE*::P*_sspE_*-*lacZ*, closed circles), the σ^G^ ^F91AY94A^ mutant (AH6539, *sigGF91AY94A* Δ*yycR:*:P*_sspE_*-*cfp* Δ*sspE*::P*_sspE_*-*lacZ,* open circles). Samples were collected every hour during stationary phase in DSM and assayed for β-galactosidase activity (shown in Miller Units). Panel C shows the immunoblot analysis of σ^G^ accumulation during sporulation in a wild-type background (AH6567, Δ*yycR:*:P*_sspE_*-*cfp* Δ*sspE*::P*_sspE_*-*lacZ*, panel E), and in the σ^GF91A/Y94A^ mutant (AH6539, *sigGF91AY94A* Δ*yycR:*:P*_sspE_*-*cfp* Δ*sspE*::P*_sspE_*-*lacZ*). Samples from sporulating cultures were collected at the onset of stationary phase in DSM and at hourly intervals thereafter, as indicated by the numbers above the lanes. Lanes labelled “ΔG” in all panels contain an extract prepared from a Δ*sigG* deletion mutant at hour 4, as a control for the specificity of the antibody. σ^A^ levels were monitored as a control for loading. The position of σ^G^ and σ^A^ is indicated by arrowheads.

**Figure S2.** Panel A, expression of a σ^F^-dependent P*_spoIIQ_-lacZ* fusion was monitored during sporulation in the following strains: wild type background AH3447 (Δ*spoIIQ*::P*_spoIIQ_*-*lacZ*, closed squares), and σ^GN45E^ mutant AH6610 (*sigGN45E* Δ*spoIIQ*::P*_spoIIQ_*-*lacZ,* open squares). Samples from stationary phase cells in DSM were collected every hour and assayed for β-galctosidase activity (shown in Miller Units). Panel B, SpoIIAB inhibits the activity of σ^G^ or σ^GN45E^ in vivo*. B. subtilis* strains carrying a P*_sspE_*-*lacZ* fusion as a reporter of σ^G^-activity, a P*_spac_*-*spoIIAB* fusion to allow the inducible production of SpoIIAB, and xylose-inducible fusions to *sigG* (AH2492; P*_xylA_*-*sigG*), *sigGE156K* (AH2493, P*_xylA_*-*sigGE156K*) or *sigGN45E* (AH6556, P*_xylA_*-*sigGN45E*) were grown in LB in the presence (1 mM) or in the absence of IPTG. Samples were taken at indicated times during growth and assayed for β-galactosidase production. The graph shows the percentage of the activities of σ^G^ (black bars), σ^GE156K^ (white bars) or σ^GN45E^ (gray bars) found in the absence and in the presence of IPTG. Panel C documents the accumulation of σ^G^ and σ^GN45E^ during stationary phase in DSM using an anti-σ^G^ antibody. The accumulation of σ^G^ and σ^GN45E^ was examined in a wild-type background and in a Δ*csfB* mutant. Samples were collected at the onset of stationary phase in DSM and at hourly intervals thereafter, as indicated by the numbers above the lanes. The lanes labeled “ΔG” in all panels contain an extract from a *sigG* deletion prepared at hour 4, as a control for the specificity of the antibody. σ^A^ levels were monitored as a loading control. The position of σ^G^ and σ^A^ is indicated by arrowheads.

**Figure S3. Time-lapse microscopy of P*_sspE_*-*cfp***. Sporulating cells were incubated on agarose pads at 30ºC. Images sequences were initiated 2 hours after the onset of sporulation in DSM and the first image was set to t=0 min. Minutes after t=0 is indicated in the lower right corner of each CFP image. The upper panels show phase contrast; the lower panels show whole cell CFP. The arrow indicates a cell with whole cell CFP accumulation.

**Figure S4.** Panel A: The *B. subtilis* CsfB protein shows structural similarity to several proteins containing zinc finger domains. Several of these proteins belong to the family of nuclear hormone receptor transcriptional regulators, with one of the most significant hits to the human vitamin D3 receptor protein (pdb code: 1kb2). The critical Cys are shown in blue (numbering is from the beginning of the sequences shown); other identical (yellow) or conserved residues (green) are also highlighted. Panel B: an *E. coli* strain expressing a C-terminal fusion of the *Strep* II tag to CsfB under the control of the T7*lac* promoter was grown in mininal medium in the presence of iron (lanes 1-3) or zinc (lanes 4-6) and induced with 1 mM IPTG for 2 hours. Lanes are as follows: 1 and 4, total extract; 2 and 5, insoluble fraction; 3 and 6, soluble fraction. Panel C depicts the induction of CsfB-*Strep* II tag by an auto-induction regime, and its purification. A strain with an “empty” vector is used as a control for the auto-induction (lanes 1, 3, and 5). Lanes are as follows: 1 and 2, total extract; 3 and 4, insoluble fraction; 5 and 6, soluble fraction; 7, protein (CsfB-*Strep* II tag) purified after a after a streptavidin affinity column. Panel D depicts the time-course of oxidant-induced (1 mM H_2_O_2_) zinc release by purified CsfB, as monitored after reaction with 4-(2-pyridylazo) resorcinol (PAR), by measuring the OD at 500 nm. No zinc is released when the protein is kept reduced in the presence of DTT.

**Figure S5.** Panel A: growth curves for strain AH6689 in LB containing the indicated xylose concentrations. Panel B: expression of P*_sspE_-cfp* and *csfB-yfp* was monitored in the same cells, by fluorescence microscopy, at the onset of stationary phase in LB. The strain used (AH6689) additionally carries a deletion of the *sigG* gene, and a second copy of the wild type *sigG* gene under the control of the xylose inducible P*_xylA_* promoter inserted at the *amyE* locus. Cells were were grown in the presence of different concentrations of xylose, as indicated (NB: images obtained in the presence of 0.0001 % xylose are not represented for simplicity). Scale bar, 2 µm. Panel C: quantitative analysis of CFP and YFP expression for the AH6689 strain (as in panel B), at the xylose concentrations indicated in panel A. The top graph shows the correlation between the YFP (*csfB-yfp*) and CFP (P*_sspE_-cfp*) signals for the various concentrations of xylose. The middle and bottom graph show a cumulative frequency distribution of the YFP and CFP signals across the population. In all three panels the fluorescence intensity is shown in arbitrary units; 100 cells were scored. The legend applies to all three graphs.

**Figure S6**. Panel A shows the crystal structure of the *Thermus aquaticus* σ^70^-containing RNA polymerase holoenzyme (RNAP), drawn with PyMol ([www.pymol.org](http://www.pymol.org/)) from the coordinates reported by Murakami *et al.* (2002). The two α subunits are shown one in blue, the other in purple, β is colored grey, β´ is shown in green, and σ^70^ in yellow. The region encircled, part of the σ70/β´ interface, is magnified in Panel B. Here, the contact between E189 in the σ^70^ subunit and R159 in β´ can be clearly seen. Other amino acids located close to the E189 residue and contributing to the interaction are represented, as are the distances (in Å) between them. Residue E189 is equivalent to N45 in σ^G^ and E39 in σ^F^.

Tables

**Table S1 - *Bacillus subtilis* strains used in this work.**

| **Strain** | Relevant Genotype | Origin |
| --- | --- | --- |
| MB24 | *trpC2 metC3* | Laboratory stock |
| AH1042 | *trpC2 metC3* Δ*sspE::*P*_sspE_-lacZ* | “ |
| AH2357 | *trpC2 metC3* Δ*sigF::erm* | “ |
| AH2452 | *trpC2* Δ*sigG* Δ*sspE::*P*_sspE_-lacZ* | [1] |
| AH2492 | *trpC2* Δ*sigG* Δ*sspE::*P*_sspE_-lacZ*  Δ*amyE::*P*_xylA_-sigG* Δ*thrC::*P*_spac_-spoIIAB* | « |
| AH2493 | *trpC2* Δ*sigG* Δ*sspE::*P*_sspE_-lacZ*  Δ*amyE::*P*_xylA_-sigGE156K* Δ*thrC::*P*_spac_-spoIIAB* | « |
| AH3447 | *trpC2 metC3 spoIIQΩspoIIQ-lacZ* | “ |
| AH3795 | *trpC2* Δ*sigG* | [1] |
| AH6513 | *trpC2* Δ*csfB::km* Δ*sigG* Δ*sspE::*P*_sspE_-lacZ* Δ*yycR::*P*_sspE_-cfp* | This work |
| AH6539 | *trpC2 sigGF91AY94A* Δ*yycR::*P*_sspE_-cfp* Δ*sspE::*P*_sspE_-lacZ* | « |
| AH6556 | *trpC2* Δ*sigG* Δ*sspE::*P*_sspE_-lacZ*  Δ*amyE::*P*_xylA_-sigGN45E* Δ*thrC::*P*_spac_-spoIIAB* | « |
| AH6566 | *trpC2* Δ*sigG* Δ*sspE::*P*sspE-lacZ* Δ*yycR::*P*_sspE_-cfp* | [11] |
| AH6567 | *trpC2* Δ*sspE::*P*_sspE_-lacZ* Δ*yycR::*P*_sspE_-cfp* | This work |
| AH6568 | *trpC2 metC3* Δ*csfB::km* Δ*sspE::*P*_sspE_-lacZ* Δ*yycR::*P*_sspE_-cfp* | « |
| AH6574 | *trpC2 sigGN45E* Δ*sspE::*P*_sspE_-lacZ* Δ*yycR::*P*_sspE_-cfp* | « |
| AH6575 | *trpC2* Δ*csfB::km sigGN45E* Δ*sspE::*P*_sspE_-lacZ* Δ*yycR::*P*_sspE_-cfp* | « |
| AH6581 | *trpC2 metC3* Δ*spoIIIJ::sp* Δ*sspE::*P*_sspE_-lacZ* Δ*yycR::*P*_sspE_-cfp* | « |
| AH6584 | *trpC2 metC3* Δ*csfB::km* Δ*spoIIIJ::sp* Δ*sspE::*P*_sspE_-lacZ* Δ*yycR::*P*_sspE_-cfp* | « |
| AH6587 | *trpC2* Δ*spoIIIJ::sp sigGN45E* Δ*sspE::*P*_sspE_-lacZ* Δ*yycR::*P*_sspE_-cfp* | « |
| AH6608 | *trpC2 metC3* Δ*csfB::km* Δ*sigG* Δ*amyE::csfB-gfp* | « |
| AH6609 | *trpC2 sigGN45A* Δ*sspE::*P*_sspE_-lacZ* Δ*yycR::*P*_sspE_-cfp* | « |
| AH6610 | *trpC2 sigGN45E spoIIQΩspoIIQ-lacZ* | « |
| AH6614 | *trpC2* Δ*yycR::*P*_sspE_-cfp* Δ*csfB::csfB-yfp* | « |
| AH6617 | *trpC2 sigGN45E* Δ*yycR::*P*_sspE_-cfp* Δ*csfB::csfB-yfp* | « |
| AH6625 | *trpC2 sigGN45E* Δ*yycR::*P*_sspE_-cfp* Δ*sspE::*P*_sspE_-lacZ sigF::erm* | « |
| AH6626 | *trpC2 sigGN45E* Δ*yycR::*P*_sspE_-cfp* Δ*sspE::*P*_sspE_-lacZ spo0A::neo* | « |
| AH6633 | *trpC2 sigGK5D* Δ*sspE::*P*_sspE_-lacZ* Δ*yycR::*P*_sspE_-cfp* | « |
| AH6634 | *trpC2 sigGC9K* Δ*sspE::*P*_sspE_-lacZ* Δ*yycR::*P*_sspE_-cfp* | « |
| AH6635 | *trpC2 sigGD10-11* Δ*sspE::*P*_sspE_-lacZ* Δ*yycR::*P*_sspE_-cfp* | « |
| AH6636 | *trpC2 sigGD12K* Δ*sspE::*P*_sspE_-lacZ* Δ*yycR::*P*_sspE_-cfp* | « |
| AH6637 | *trpC2 sigGT13N* Δ*sspE::*P*_sspE_-lacZ* Δ*yycR::*P*_sspE_-cfp* | « |
| AH6638 | *trpC2 sigGL16N* Δ*sspE::*P*_sspE_-lacZ* Δ*yycR::*P*_sspE_-cfp* | « |
| AH6639 | *trpC2 sigGV18Q* Δ*sspE::*P*_sspE_-lacZ* Δ*yycR::*P*_sspE_-cfp* | « |
| AH6640 | *trpC2 sigGN21D* Δ*sspE::*P*_sspE_-lacZ* Δ*yycR::*P*_sspE_-cfp* | « |
| AH6641 | *trpC2 sigGM24V* Δ*sspE::*P*_sspE_-lacZ* Δ*yycR::*P*_sspE_-cfp* | « |
| AH6642 | *trpC2 sigGR25K* Δ*sspE::*P*_sspE_-lacZ* Δ*yycR::*P*_sspE_-cfp* | « |
| AH6643 | *trpC2 sigGF28I* Δ*sspE::*P*_sspE_-lacZ* Δ*yycR::*P*_sspE_-cfp* | « |
| AH6644 | *trpC2 sigGD33N* Δ*sspE::*P*_sspE_-lacZ* Δ*yycR::*P*_sspE_-cfp* | « |
| AH6645 | *trpC2 sigGD37Q* Δ*sspE::*P*_sspE_-lacZ* Δ*yycR::*P*_sspE_-cfp* | « |
| AH6646 | *trpC2 sigGS38Q* Δ*sspE::*P*_sspE_-lacZ* Δ*yycR::*P*_sspE_-cfp* | « |
| AH6647 | *trpC2 sigGK42L* Δ*sspE::*P*_sspE_-lacZ* Δ*yycR::*P*_sspE_-cfp* | « |
| AH6648 | *trpC2 sigGV44I* Δ*sspE::*P*_sspE_-lacZ* Δ*yycR::*P*_sspE_-cfp* | « |
| AH6649 | *trpC2 sigGG46K* Δ*sspE::*P*_sspE_-lacZ* Δ*yycR::*P*_sspE_-cfp* | « |
| AH6650 | *trpC2 sigGL48M* Δ*sspE::*P*_sspE_-lacZ* Δ*yycR::*P*_sspE_-cfp* | « |
| AH6651 | *trpC2 sigGL52W* Δ*sspE::*P*_sspE_-lacZ* Δ*yycR::*P*_sspE_-cfp* | « |
| AH6652 | *trpC2 sigGN59L* Δ*sspE::*P*_sspE_-lacZ* Δ*yycR::*P*_sspE_-cfp* | « |
| AH6653 | *trpC2 sigGE63Y* Δ*sspE::*P*_sspE_-lacZ* Δ*yycR::*P*_sspE_-cfp* | « |
| AH6654 | *trpC2 sigGY64E* Δ*sspE::*P*_sspE_-lacZ* Δ*yycR::*P*_sspE_-cfp* | « |
| AH6655 | *trpC2 sigGV65P* Δ*sspE::*P*_sspE_-lacZ* Δ*yycR::*P*_sspE_-cfp* | « |
| AH6656 | *trpC2 sigGV71I* Δ*sspE::*P*_sspE_-lacZ* Δ*yycR::*P*_sspE_-cfp* | « |
| AH6657 | *trpC2 sigGM77L* Δ*sspE::*P*_sspE_-lacZ* Δ*yycR::*P*_sspE_-cfp* | « |
| AH6658 | *trpC2 sigGN82K* Δ*sspE::*P*_sspE_-lacZ* Δ*yycR::*P*_sspE_-cfp* | « |
| AH6678 | *trpC2 sigGF91AY94A* Δ*yycR::*P*_sspE_-cfp* Δ*csfB::csfB-yfp* | « |
| AH6679 | *trpC2 sigGN45EF91AY94A* Δ*yycR::*P*_sspE_-cfp* Δ*csfB::csfB-yfp* | « |
| AH6680 | *trpC2 sigGN45E* Δ*yycR::*P*_sspE_-cfp* Δ*csfB::csfB-yfp sigF::erm* | « |
| AH6686 | *trpC2 metC3* Δ*sigF::erm* *ΔthrC:*:P*_spac_*-*csfB* Δ*amyE*::*spoIIAABCwt* | « |
| AH6687 | *trpC2 metC3* Δ*sigF::erm* *ΔthrC:*:P*_spac_*-*csfB* Δ*amyE*::*spoIIAABC E39N* | « |
| AH6688 | *trpC2* Δ*sigG* Δ*yycR::*P*_sspE_-cfp* Δ*csfB::csfB-yfp* Δ*amyE::P_xylA_-sigGE156K* | « |
| AH6689 | *trpC2* Δ*sigG* Δ*yycR::*P*_sspE_-cfp* Δ*csfB::csfB-yfp* Δ*amyE::P_xylA_-sigGwt* | « |
| AH6690 | *trpC2* Δ*sigG* Δ*yycR::*P*_sspE_-cfp* Δ*csfB::csfB-yfp* Δ*amyE::P_xylA_-sigGN45E* | « |
| AH6691 | *trpC2 metC3* Δ*sigF::erm* Δ*thrC:*:P*_spac_*-*csfB* *ΔamyE*::*spoIIAABCwt yuiC::*P*_yuiC_-gfp* | « |
| AH6692 | *trpC2 metC3* Δ*sigF::erm* Δ*thrC:*:P*_spac_*-*csfB* Δ*amyE*::*spoIIAABC E39N yuiC::*P*_yuiC_-gfp* | « |
| AH6698 | *trpC2* Δ*sigG* Δ*yycR::*P*_sspE_-cfp* Δ*csfB::csfB-yfp* Δ*amyE::*P*_xylA_sigGwt* Δ*lonA::cat* | « |
| AH6723 | *trpC2* Δ*sigG* *csfB::csfB-lacZ* Δ*amyE::P_xylA_-sigGwt* | “ |
| AH6724 | *trpC2* Δ*sigG* *csfB::*P*_csfB_-lacZ* Δ*amyE::P_xylA_-sigGwt sigF::erm* | “ |
| AH6789 | *trpC2* Δ*sigG* Δ*yycR::*P*_sspE_-cfp* Δ*csfB::csfB-yfp* Δ*amyE*::P*_xylA_-sigGN45E/E156K* | « |
| BTD2633 | Δ*yycR::*P*_sspE_-cfp* | D. Rudner |
| JOB20 | *trpC2 metC3* Δ*spoIIIJ::sp* | [12] |
| MO3632 | Δ*csfB::km* | P. Stragier |
| SL14127 | Δ*thrC:*:P*_spac_*-*csfB* Δ*amyE::*P*_gerE_-lacZ* | P. Piggot |

Table S2 – Oligonucleotide primers used in this work.

| ***Primer*** | Sequence (5’ 🡪 3’) |
| --- | --- |
| *csfB12D* | CGCCGCCAAGCAAGCTTAAACCCAGC |
| *csfB191D* | GGTGGAGACCATGGACGAAACAG |
| *csfB681D* | CCATGAATTCATAGACCTGAAAAGGTC |
| *csfB480R* | ATTATTATTGTCGACTACGTTCAATCC |
| *csfB1146R* | CTCAGGATCCTTAGCATTTCTGCATTTTGC |
| *csfBgfpR* | GTTCTTCTGGTTTACTGTTGTTGTTGTTTGAATATAATGGCGGTGTATG |
| *csfBstrepR* | CGGGATCCTTATTTTTCGAACTGCGGGTGGCTCCAAGCGCTTGAATATAATGGCGGTGTATG |
| *gfp30D* | AGTAAAGGAGAAGAACTTTTCACTGGAG |
| *gfpR* | GGCGGATCCTTATTTGTATAGTTCATCCATGC |
| *yfpR* | GCATGCCTGCAGGTCTGGAC |
| *sigF493D* | GATTTGCCATGGATGTGGAGG |
| *sigF219D* | GAAGGATCCGAAGAAAGCCGGG |
| *sigF1318R* | GACCATGAATTCCCACGCTTTTGC |
| *sigF2032R* | GATTAAGCTTCTCATTCATCCGCTCG |
| *sigFE39ND* | GAGACCTCCTCATAAACAAAAACATGCGTC |
| *sigFE39NR* | GACGCATGTTTTTGTTTATGAGGAGGTCTC |
| *sigG2016D* | GGGAAAACCATGGCGAGAAATAAAGTCG |
| *sigG2862R* | CCATCCAGAATTCAATAGAAAAGCC |
| *sigG2964R* | GCAAAATCCTCGAGGTCCTCCTCTTTATTGCC |
| *sigGK5DD* | GTGTCGAGAAATGATGTCGAAATCTGCGGG |
| *sigGK5DR* | CCCGCAGATTTCGACATCATTTCTCGACAC |
| *sigGC9KD* | GAAATAAAGTCGAAATCAAAGGGGTGGATACCTCC |
| *sigGC9KR* | GGAGGTATCCACCCCTTTGATTTCGACTTTATTTC |
| *sigGD10-11D* | GATACCTCCAAATTACCAGTACTC |
| *sigGD10-11R* | GCAGATTTCGACTTTATTTCTCG |
| *sigGD12KD* | CGAAATCTGCGGGGTGAAAACCTCCAAATTACC |
| *sigGD12KR* | GGTAATTTGGAGGTTTTCACCCCGCAGATTTCG |
| *sigGT13ND* | CTGCGGGGTGGATAACTCCAAATTACCAG |
| *sigGT13NR* | CTGGTAATTTGGAGTTATCCACCCCGCAG |
| *sigGL16ND* | GGTGGATACCTCCAAAAATCCAGTACTCAAGAATG |
| *sigGL16NR* | CATTCTTGAGTACTGGATTTTTGGAGGTATCCACC |
| *sigGV18QD* | CCTCCAAATTACCACAACTCAAGAATGAAGAG |
| *sigGV18QR* | CTCTTCATTCTTGAGTTGTGGTAATTTGGAGG |
| *sigGN21DD* | CCAGTACTCAAGGATGAAGAGATGAGAAAGC |
| *sigGN21DR* | GCTTTCTCATCTCTTCATCCTTGAGTACTGG |
| *sigGM24VD* | CTCAAGAATGAAGAGGTGAGAAAGCTGTTTAGGC |
| *sigGM24VR* | GCCTAAACAGCTTTCTCACCTCTTCATTCTTGAG |
| *sigGR25KD* | GAATGAAGAGATGAAAAAGCTGTTTAGGC |
| *sigGR25KR* | GCCTAAACAGCTTTTTCATCTCTTCATTC |
| *sigGF28ID* | GAGATGAGAAAGCTGATTAGGCAGCTGCAGG |
| *sigGF28IR* | CCTGCAGCTGCCTAATCAGCTTTCTCATCTC |
| *sigGD33ND* | GTTTAGGCAGCTGCAGAATGAAGGCGATGATTCAGC |
| *sigGD33NR* | GCTGAATCATCGCCTTCATTCTGCAGCTGCCTAAAC |
| *sigGD37QD* | GCAGGATGAAGGCGATCAATCAGCAAGAGAAAAG |
| *sigGD37QR* | CTTTTCTCTTGCTGATTGATCGCCTTCATCCTGC |
| *sigGS38QD* | GGATGAAGGCGATGATCAAGCAAGAGAAAAGC |
| *sigGS38QR* | GCTTTCTCTTGCTTGATCATCGCCTTCATCC |
| *sigGK42LD* | GATTCAGCAAGAGAATATCTTGTAAACGGGAAC |
| *sigGK42LR* | GTTCCCGTTTACAAGATATTCTCTTGCTGAATC |
| *sigGV44ID* | GCAAGAGAAAAGCTTATAAACGGGAACTTGCG |
| *sigGV44IR* | CGCAAGTTCCCGTTTATAAGCTTTTCTCTTGC |
| *sigGN45ED* | GAGAAAAGCTTGTAGAGGGGAACTTGCGTCTTC |
| *sigGN45ER* | GAAGACGCAAGTTCCCCTCTACAAGCTTTTCTC |
| *sigGN45AD* | GAGAAAAGCTTGTAGCCGGGAACTTGCGTCTTG |
| *sigGN45AR* | CAAGACGCAAGCTCCCGGCTACAAGCTTTTCTC |
| *sigGG46KD* | GAAAAGCTTGTAAACAAGAACTTGCGTCTTG |
| *sigGG46KR* | CAAGACGCAAGTTCTTGTTTACAAGCTTTTC |
| *sigGL48MD* | GTAAACGGGAACATGCGTCTTGTCTTAAGTG |
| *sigGL48MR* | CACTTAAGACAAGACGCATGTTCCCGTTTAC |
| *sigGL52WD* | GGAACTTGCGTCTTGTCTGGAGTGTCATTCAACG |
| *sigGL52WR* | CGTTGAATGACACTCCAGACAAGACGCAAGTTCC |
| *sigGN59LD* | GTCATTCAACGATTTCTTAACAGAGGGGAG |
| *sigGN59LR* | CTCCCCTCTGTTAAGAAATCGTTGAATGAC |
| *sigGE63YD* | CGATTTAATAACAGAGGGTATTATGTTGATGAC |
| *sigGE63YR* | GTCATCAACATAATACCCTCTGTAATTAAATCG |
| *sigGY64ED* | CAGAGGGGAGGAAGTTGATGAC |
| *sigGY64ER* | GTCATCAACTACCTCCCCTCTG |
| *sigGV65PD* | CAGAGGGGAGTATCCTGATGACTTATTTC |
| *sigGV65PR* | GAAATAAGTCATCAGGATACTCCCCTCTG |
| *sigGV71ID* | GACTTATTTCAAGCCGGCTGCATCGGAC |
| *sigGV71IR* | GTCCGATGCAGCCGGCTTGAAATAAGT |
| *sigGM77LD* | CTGCATCGGACTATTGAAATCCATTG |
| *sigGM77LR* | CAATGGATTTCAATAGTCCGATGCAG |
| *sigGN82KD* | GAATCCATTGATAAATTTGACCTAAGCCAC |
| *sigGN82KR* | GTGGCTTAGGTCAAATTTATCAATGGATTTC |
| *sigGF91AY94AD* | GCCACAATGTTAAGGCTTCAACAGCCGCTGTACCTATGATTATCGG |
| *sigGF91AY94AR* | CCGATAATCATAGGTACAGCGGCTGTTGAAGCCTTAACATTGTGGC |
| *spoIIAB189D* | GGGGGTGGATCCATGAAAAATGAAATGC |
| *spoIIAB698R* | CCTTCAGCTCGAGCGTTTTTGCCG |
| *csfB578R* | CGTATAAAGATGGATCCTCTATTCTTCTC |
